# Implementation of a national AI technology program on cardiovascular outcomes and the health system

**DOI:** 10.1038/s41591-025-03620-y

**Published:** 2025-04-04

**Authors:** Timothy A. Fairbairn, Liam Mullen, Edward Nicol, Gregory Y. H. Lip, Matthias Schmitt, Matthew Shaw, Laurence Tidbury, Ian Kemp, Jennifer Crooks, Girvan Burnside, Sumeet Sharma, Anoop Chauhan, Chee Liew, Sawan Waidyanatha, Sri Iyenger, Andrew Beale, Imran Sunderji, John P. Greenwood, Manish Motwani, Anna Reid, Anna Beattie, Justin Carter, Peter Haworth, Nicholas Bellenger, Benjamin Hudson, Jonathan Rodrigues, Oliver Watson, Vinod Venugopal, Russell Bull, Peter O’Kane, Aparna Deshpande, Gerald P. McCann, Simon Duckett, Hatef Mansoubi, Victoria Parish, Joban Sehmi, Campbell Rogers, Sarah Mullen, Jonathan Weir-McCalL

**Affiliations:** 1 Liverpool Centre for Cardiovascular Science, Liverpool Heart and Chest Hospital, Liverpool, UK; 2 Institute of Life Course and Medical Sciences, Faculty of Health and Life Sciences, University of Liverpool, Liverpool, UK; 3 Royal Brompton and Harefield Hospital, Guys and St Thomas’ NHS Trust, London, UK; 4 Department of Cardiovascular Imaging, Faculty of Life Sciences and Medicine, Kings College London, London, UK; 5 Manchester University NHS Foundation Trust, Manchester, UK; 6 Institute of Population Health, Faculty of Health and Life Sciences, University of Liverpool, Liverpool, UK; 7 Ashford and St Peters Hospital NHS Foundation Trust, London, UK; 8 Blackpool Teaching Hospitals NHS Foundation Trusts, Blackpool, UK; 9 Dudley Group NHS Foundation Trust, Birmingham, UK; 10 Frimley Health NHS Foundation Trust, Guildford, UK; 11 Great Western Hospitals NHS Foundation Trust, Swindon, UK; 12 Hull University Teaching Hospitals NHS Trust, Hull, UK; 13 Leeds Institute of Cardiovascular and Metabolic Medicine, University of Leeds and Leeds Teaching Hospitals NHS Trust, Leeds, UK; 14 Newcastle Upon Tyne Hospitals NHS Foundation Trust, Newcastle upon Tyne, UK; 15 North Tees and Hartlepool NHS Foundation Trust, Middlesbrough, UK; 16 Portsmouth Hospitals NHS Trust, Portsmouth, UK; 17 Royal Devon and Exeter Hospital NHS Trust, Exeter, UK; 18 Royal United Hospitals Bath NHS Foundation Trust, Bath, UK; 19 Sheffield Teaching Hospitals NHS Foundation Trust, Sheffield, UK; 20 United Lincolnshire Hospitals NHS Trust, Lincoln, UK; 21 University Hospital Dorset NHS Trust, Bournemouth, UK; 22 University Hospitals of Leicester NHS Trust, Leicester, UK; 23 University of Leicester, Leicester, UK; 24 University Hospitals of North Midlands NHS Trust, Stoke-On-Trent, UK; 25 University Hospitals Sussex NHS Foundation Trust, Brighton, UK; 26 West Hertfordshire Hospital NHS Trust, Watford, UK; 27 HeartFlow Inc, Mountainview, CA USA; 28 Royal Papworth Hospital NHS Foundation Trust, Cambridge, UK

**Keywords:** Computed tomography, Cardiovascular diseases

## Abstract

Coronary artery disease (CAD) is a major cause of ill health and death worldwide. Coronary computed tomographic angiography (CCTA) is the first-line investigation to detect CAD in symptomatic patients. This diagnostic approach risks greater second-line heart tests and treatments at a cost to the patient and health system. The National Health Service funded use of an artificial intelligence (AI) diagnostic tool, computed tomography (CT)-derived fractional flow reserve (FFR-CT), in patients with chest pain to improve physician decision-making and reduce downstream tests. This observational cohort study assessed the impact of FFR-CT on cardiovascular outcomes by including all patients investigated with CCTA during the national AI implementation program at 27 hospitals (CCTA *n* = 90,553 and FFR-CT *n* = 7,863). FFR-CT was safe, with no difference in all-cause (*n* = 1,134 (3.2%) versus 1,612 (2.9%), adjusted-hazard ratio (aHR) 1.00 (0.93–1.08), *P* = 0.97) or cardiovascular mortality (*n* = 465 (1.3%) versus 617 (1.1%), aHR 0.96 (0.85–1.08), *P* = 0.48), while reducing invasive coronary angiograms (*n* = 5,720 (16%) versus 8,183 (14.9%), aHR 0.93 (0.90–0.97), *P* < 0.001) and noninvasive cardiac tests (189/1,000 patients versus 167/1,000), *P* < 0.001). Implementation of an AI-diagnostic tool as part of a health intervention program was safe and beneficial to the patient pathway and health system with fewer cardiac tests at 2 years.

## Abstract (web summary)

National implementation of a computed tomographic angiography and AI-diagnostic tool, CT-derived fractional flow reserve (FFR-CT), did not provide significant benefits in reducing mortality but led to fewer invasive coronary angiograms and downstream cardiac tests.

## Main

Coronary artery disease (CAD) remains a major cause of symptoms, morbidity and death worldwide. International guidelines endorse coronary computed tomography angiography (CCTA) as a first-line investigation for patients with suspected stable symptomatic CAD, principally to diagnose or exclude CAD^1,2^. The National Institute of Health and Care Excellence (NICE) in the UK was the first to recommend this diagnostic strategy for patients with possible anginal symptoms and no known CAD^3^. This decision was controversial, as CCTA has limited ability to link the presence of obstructive CAD to symptoms such as angina, and evidence from health systems where CCTA was adopted early showed that increased inappropriate invasive coronary angiograms (ICAs) and percutaneous coronary interventions (PCIs) could be a consequence of this diagnostic pathway^4^.

To further improve the CCTA diagnostic pathway and reduce the risk of unnecessary future tests or revascularizations, NICE subsequently recommended a new artificial intelligence (AI)-augmented technology, computed tomography (CT)-derived fractional flow reserve (FFR-CT) (HeartFlow), to be used on CCTA images when there was the presence of possibly flow-limiting CAD^5^. FFR-CT utilizes CCTA raw images to generate an AI deep-learned coronary segmentation that is combined with a three-dimensional computational fluid dynamics model of coronary arterial blood flow^6,7^. This technology is dependent on high-quality imaging and close adherence to the recommended guidelines on performing CCTA to produce an accurate three-dimensional model. FFR-CT models the combined anatomy and physiology of coronary arteries to inform the interpreting physician whether any CAD seen is probably flow limiting and, thus, causing patient symptoms. Several randomized controlled trials have demonstrated the diagnostic accuracy of FFR-CT and its potential to transform the patient chest pain pathway by reducing unnecessary ICA at a modeled neutral cost^8,9^.

NICE considered the evidence for the use of the AI technology based on its clinical effectiveness, system impact on the National Health Service (NHS) and likely cost–benefit, stating that FFR-CT had high accuracy, would reduce unnecessary invasive tests and save the NHS £9.1 million per year^5^. Despite this, the uptake of FFR-CT in clinical practice was limited due to the cost of the technology, hospital funding constraints and a ‘black box’ component to the external AI analysis^10^. The challenges to implementing AI decision support systems have been recognized globally, including at the 2024 Responsible AI for Social and Ethical Healthcare conference^11^.

In an attempt to address these obstacles, NHS England launched an innovation and technology program to centrally fund the use of new AI technologies within the national healthcare system. FFR-CT was chosen with the aim of improving patient care by avoiding unnecessary downstream tests, while benefiting an overburdened healthcare system. This retrospective quasi-experimental observational cohort study was designed to examine whether this specific health intervention of implementing an AI tool (FFR-CT) in a national health system was clinically useful and safe by improving clinical and health system outcomes^12,13^.

## Results

### Patient characteristics

**Fig. 1:**
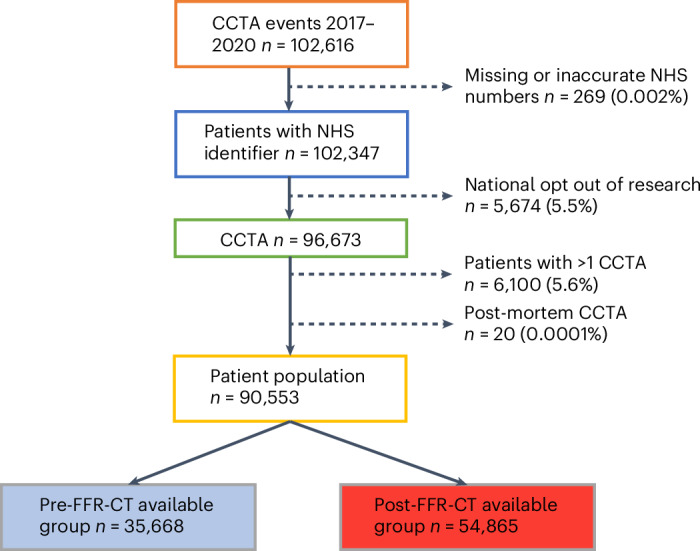
Flow diagram of the 3-year coronary computed tomography angiography (CCTA) data. Data provided by the 27 NHS England hospitals with subsequent identification of patient numbers, repeat tests and patients excluded due to withdrawal of consent (national ‘Opt out of research’ database).

**Extended Data Fig. 1:**
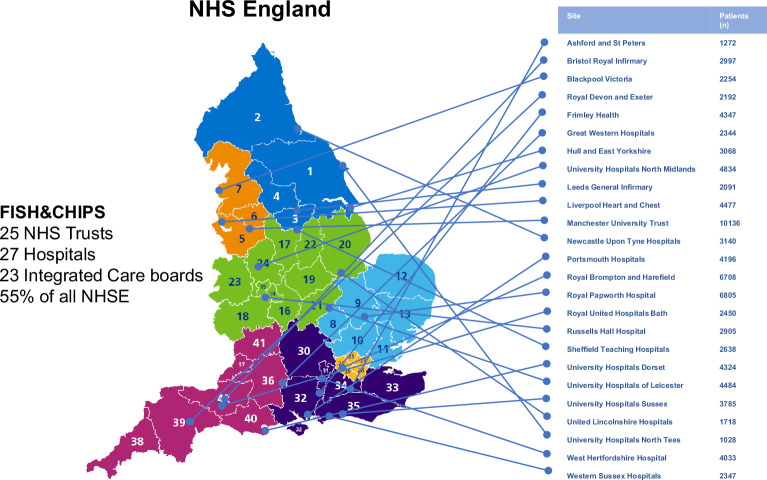
English hospital site map. A map of NHS England with the 25 different NHS Trusts plotted, their corresponding Integrated Care Board (ICB) and number of patients (n) contributed to the study.

**Extended Data Fig. 2:**
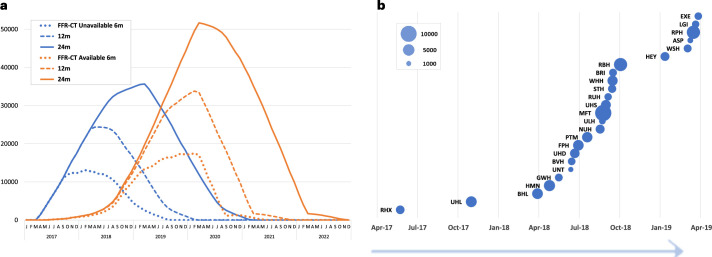
Patient recruitment and site onboarding for the AI technology. **a** Patient recruitment and drop out for 6 months, 12 months and 24 month time points: Categorized as pre (blue line) or post (red line) FFR-CT availability. All 27 sites provided patient data from April 2017 – April 2020 with 2 sites (UHD and BRI) providing data from April 2017-December 2020. **b** Site FFR-CT availability: Introduction of FFR-CT at a site level was used to define whether the CCTA was performed before or after FFR-CT was made available at their site. The bubbles represent the time that FFR-CT was made available for clinical use and the number of CCTA scans performed at each site over the study recruitment time period (April 2017-December 2020).

Between April 2017 and December 2020, 102,616 CCTAs were performed at 27 sites across a widespread geographic distribution, at secondary and tertiary hospitals in England that are representative of NHS clinical practice (Extended Data Figs. 1 and 2). There were 289 (0.28%) patients without an NHS number, 5,674 (5.5%) patients withdrew their consent, 6,100 (5.9%) CCTA were repeat studies on the same patient during the study period and 20 (0.0001%) patients had a post-mortem CCTA. The final study population of 90,553 patients consisted of 35,688 who had undergone CCTA before the introduction of FFR-CT, and 54,865 CCTA after FFR-CT was available at their hospital (Fig. 1). The mean age was 58 ± 13 years, 48.1% female, with varied ethnicity (78.7% white British or Irish, 2.2% black, 1.4% mixed race, 8.2% Asian, 2.4% other, 7.1% unstated).

The median follow-up for the total population was 1,211 (interquartile range (IQR) 535) days, with 98.1% (*n* = 88,842) completing 2 years follow-up. The cohorts were well matched for demographic and cardiovascular disease risk factors (Table 1) with clinically small but statistically significant differences in patients’ age, hypertension, heart failure, valve disease, chronic obstructive pulmonary disease (COPD) and chronic kidney disease. Among 54,865 CCTA patients who had FFR-CT testing available to their hospital, 7,863 (14.1%) went on to undergo FFR-CT analysis. This cohort were older (63 (IQR 55–71) years), with greater cardiovascular risk factors compared with the total population as they were selected for further testing on the basis of the presence of CAD (Supplementary Table 1).

**Table 1:** Patient characteristics of the study cohorts at baseline

	FFR-CT unavailable	FFR-CT available	*P* value
	*n* (%)	*n* (%)	
	35,688 (39.4)	54,865 (60.6)	
**Demographics**			
Age at index CT scan (years)	58 (49, 67)	59 (50, 68)	<0.0001
Female	17,141 (48.0)^a^	26,393 (48.2)^a^	0.59
Type 1 diabetes mellitus	454 (1.3)	707 (1.3)	0.84
Type 2 diabetes mellitus	4,869 (13.6)	7,516 (13.7)	0.82
Hyperlipidemia	8,397 (23.5)	12,724 (23.2)	0.24
Hypertension	14,788 (41.4)	21,905 (39.9)	<0.0001
Angina	6,457 (18.1)	9,843 (17.9)	0.57
Myocardial Iifarction	1,597 (4.5)	2,355 (4.3)	0.19
Valve disorder	3,667 (10.3)	4,890 (7.9)	0.03
Heart failure	4,014 (11.2)	5,362 (9.8)	<0.0001
TIA	341 (1.0)	454 (0.8)	0.048
Cerebral infarction	681 (1.9)	952 (1.7)	0.06
Atherosclerosis	520 (1.5)	788 (1.4)	0.79
Aortic aneurysm	1,015 (2.8)	1,475 (2.7)	0.16
COPD	3,597 (10.1)	4,401 (9.3)	0.0038
Kidney disease	2,556 (7.2)	3,496 (6.4)	<0.001
**Medications**^**b**^	***n*** = **34,554**	***n*** = **51,602**	
ACE inhibitors	7,635 (22.1)	11,502 (22.3)	0.50
Antiplatelets	10,282 (29.8)	15,592 (30.2)	0.15
Beta blockers	10,313 (29.9)	16,066 (31.1)	<0.0001
Calcium channel blockers	6,239 (18.1)	9,887 (19.2)	<0.0001
Statins	16,960 (49.1)	26,434 (51.2)	<0.0001
Vitamin K antagonist	1,096 (3.2)	1,167 (2.3)	<0.0001
Factor Xa	2,794 (8.1)	4,723 (9.2)	<0.0001
Lipid-lowering medications	699 (2.0)	1,266 (2.5)	<0.0001

Data are presented as number and percentage or number and 95% CI or IQR.

Wilcoxon–Mann–Whitney rank-sum and chi-square tests were used with a two-sided *P* value <0.05 considered significant.

^a^Among these patients, 0.14% not specified as female or male.

^b^Prescribed medications per patient over 2 years.

TIA, transient ischemic attack; ACE, angiotensin-converting enzyme.

Diagnostic codes (ICD) from the HES were used to determine patient comorbidities.

### All-cause mortality and cardiovascular outcomes

There were 2,746 deaths, of which 1,082 were cardiovascular, 1,129 myocardial infarction (MI) and 13,903 ICA performed at 2 years. The 90-day, 1-year and 2-year numbers (%) for each group are reported in Table 2.

**Table 2:** Event rates of the primary clinical outcomes (all-cause death, cardiovascular death, MI and ICA without revascularization)

	90 days		1 year		2 years					
	FFR-CT unavailable (*n* = 35,688)	FFR-CT available (*n* = 54,865)	FFR-CT unavailable (*n* = 35,667)	FFR-CT available (*n* = 53,174)	FFR-CT unavailable (*n* = 35,667)	FFR-CT available (*n* = 53,174)	Unadjusted HR (95% CI) *α*	*P* value	aHR (95% CI) *β*	*P* value
All-cause mortality	209 (0.59)	303 (0.55)	613 (1.72)	869 (1.58)	1,134 (3.17)	1,612 (2.94)	0.923 (0.856–0.996)	0.04	1.00 (0.93–1.08)	0.97
Cardiovascular mortality	118 (0.33)	167 (0.30)	291 (0.82)	399 (0.73)	465 (1.30)	617 (1.12)	0.863 (0.765–0.973)	0.02	0.96 (0.85–1.08)	0.48
MI^a^	120 (0.34)	200 (0.37)	281 (0.79)	474 (0.86)	425 (1.19)	704 (1.28)	1.082 (0.959–1.220)	0.20	1.18 (1.05, 1.34)	0.006
Revascularization (PCI and CABG)	1,196 (3.36)	2,001 (3.66)	2,352 (6.59)	3,743 (6.82)	2,603 (7.29)	4,181 (7.62)	1.048 (0.998–1.100)	0.06	1.06 (1.01, 1.11)	0.02
PCI	971 (2.73)	1,675 (3.06)	1,736 (4.89)	2,872 (5.26)	1,912 (5.35)	3,161 (5.76)	1.079 (1.019–1.142)	0.008	1.09 (1.03, 1.15)	0.002
CABG	225 (0.63)	326 (0.60)	616 (1.74)	871 (1.59)	691 (1.94)	1,020 (1.86)	0.959 (0.870–1.056)	0.39	1.01 (0.91, 1.11)	0.89
All ICA performed	3,241 (9.1)	4,511 (8.2)	5,265 (14.8)	7,444 (13.6)	5,720 (16.0)	8,183 (14.9)	0.924 (0.893–0.956)	<0.001	0.93 (0.90, 0.97)	<0.001
ICA no revascularization	2,045 (5.7)	2,510 (4.6)	2,913 (8.2)	3,701 (6.7)	3,117 (8.7)	4,002 (7.3)	0.828 (0.790–0.868)	<0.001	0.84 (0.80, 0.88)	<0.001
Revascularization ratio	40.27	47.53	47.96	52.83	48.36	52.94		<0.001		

*α* Cox-proportional hazards for primary outcomes at 2 years; *β* covariates adjusted for baseline prognostic differences (age, heart failure, hypertension, valve disease, COPD and kidney disease).

^a^Day 0 and day 1 events excluded.

**Fig. 2:**
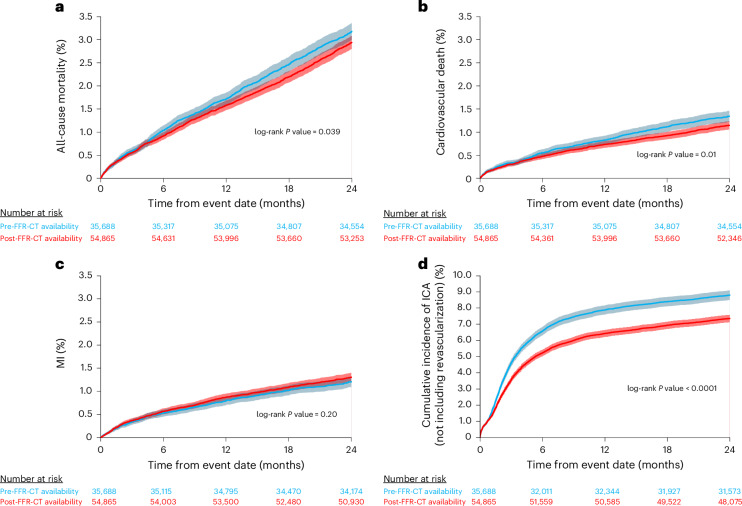
Kaplan–Meier charts of the cumulative incidence of the individual primary objectives over 2 years after index CCTA. **a**–**c**, The incidence of all-cause death (**a**), cardiovascular death (**b**) and MI (**c**) rates. **d**, The incidence of ICA without subsequent revascularization. The shaded areas indicate the 95% CIs.

There was lower all-cause (*n* = 1,134 (3.2%) versus 1,612 (2.9%), hazard ratio (HR) 0.92 (0.856–0.996), *P* = 0.04) and cardiovascular mortality (*n* = 465 (1.3%) versus 617 (1.1%), HR 0.86 (0.765–0.973), *P* = 0.02) rates observed in the FFR-CT available group at 2 years compared with the unavailable group (Fig. 2). There was no significant difference in MI events (*n* = 425 (1.2%) versus 704 (1.3%), HR 1.08 (0.96–1.22), *P* = 0.24). Rates of all ICA inclusive of those progressing to revascularization (*n* = 5,720 (16.0%) versus 8,183 (14.9%), HR 0.92 (0.89–0.96), *P* < 0.001) and ICA with no subsequent revascularization (*n* = 3,117 (8.7%) versus 4,002 (7.3%), HR 0.83 (0.79–0.87), *P* < 0.001) were significantly lower in the FFR-CT available group.

**Extended Data Fig. 3:**
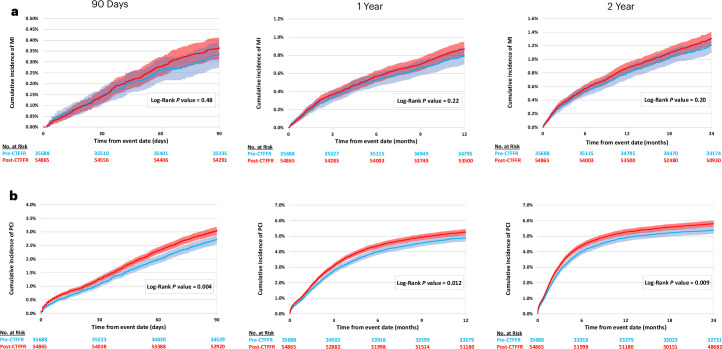
Event curves for myocardial infarction and percutaneous intervention at different time points. **a** Myocardial Infarction (MI) events and **B**. Percutaneous Intervention (PCI) rates at 90 days, 1 year and 2 years. Cox proportional hazards univariate analysis (unadjusted) p values. The shaded areas indicate the 95% CIs.

**Extended Data Fig. 4:**
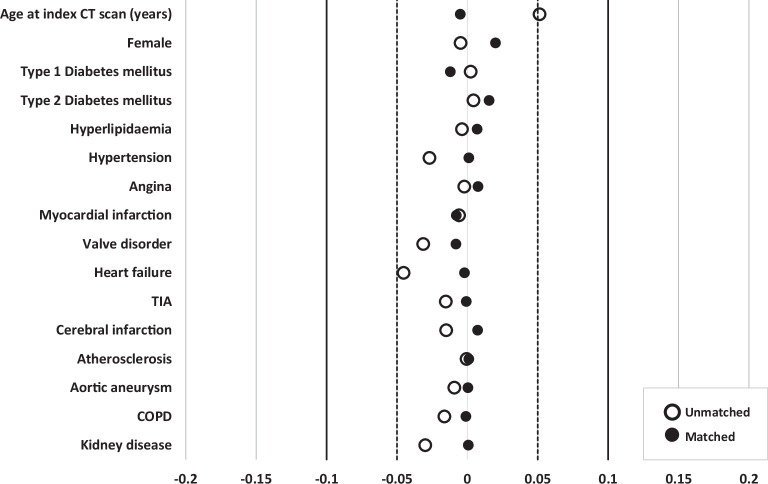
Propensity Score Matching balancing. Propensity Score Matching covariate balancing pre and post matching using a standard mean difference of <0.05.

**Extended Data Fig. 5:**
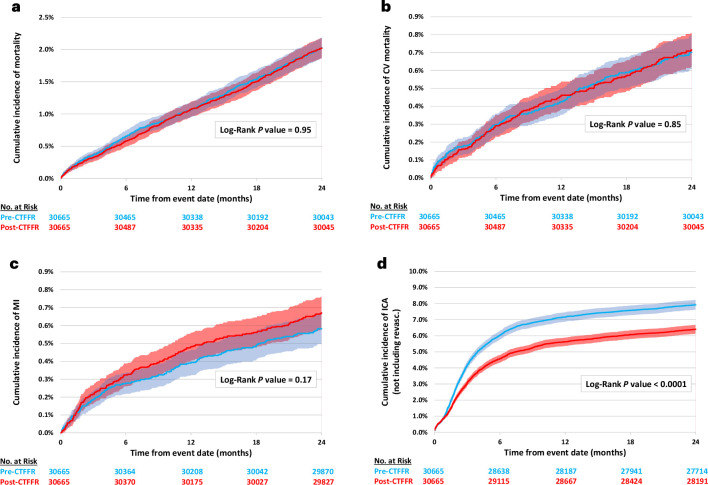
Event curves for the primary outcomes of the Propensity Matched Scoring population. **a**. All-cause death, **b**. Cardiovascular death, **c**. Myocardial infarction, **d**. ICA with no revascularization. Kaplan-Meier (KM) charts of the cumulative incidence of the individual primary objectives over 2 years post index CCTA on the Propensity Matched population (n = 30,665 in each group). Blue represents pre FFR-CT availability. Red line represents post FFR-CT availability. The shaded areas indicate the 95% CIs.

Nonbalanced prognostic factors at baseline were entered into a multivariable Cox-regression model (age, hypertension, heart failure, COPD, valve disease and chronic kidney disease). Once adjusted for baseline differences in co-morbidities, there was no significant difference in all-cause (adjusted HR (aHR) 1.00 (0.93–1.08), *P* = 0.97) or cardiovascular mortality (aHR 0.96 (0.85–1.08), *P* = 0.48) risk between the FFR-CT available and FFR-CT unavailable groups at 2 years. Adjusted risk of MI was higher in the FFR-CT available group (aHR 1.18 (1.05–1.34), *P* = 0.006) (Table 2 and Extended Data Fig. 3). The risk reduction in all ICA (aHR 0.93 (0.90–0.97), *P* < 0.001) and ICA without revascularization (aHR 0.84 (0.80–0.88), *P* < 0.001) remained after covariate adjustment (Table 2) and sensitivity analysis (Supplementary Table 2). Assessment of the proportional hazards assumption by testing for a zero slope in the scaled Schoenfeld residuals for each Cox model showed proportionality for all outcomes. Propensity score matching (PSM) resulted in two cohorts of 30,665 patients (FFR-CT unavailable and FFR-CT available) (Extended Data Fig. 4 and Supplementary Table 3). The PSM analysis showed that all-cause, cardiovascular death and MI at 2 years did not significantly differ (*P* = 0.95, *P* = 0.85 and *P* = 0.17) between groups. Lower all ICA and ICA without revascularization was still observed in the FFR-CT available cohort compared with the unavailable cohort (*P* < 0.005 and *P* < 0.001) (Extended Data Fig. 5).

**Fig. 3:**
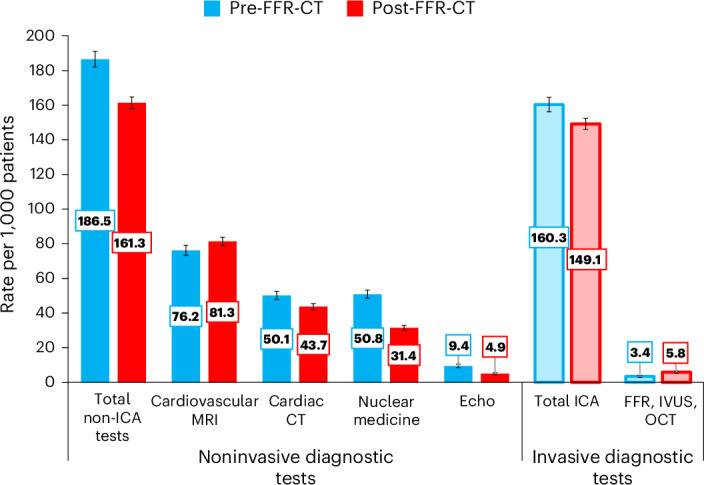
The number of cardiac diagnostics performed as a second-line test within 2 years of the index CCTA scan (rate per 1,000 patients). Subcategorized into noninvasive tests (cardiovascular MRI, cardiac CT, nuclear medicine (positron emission tomography and radionuclide imaging) and echocardiography (excluding transthoracic echocardiography)) and invasive tests (total ICA and intracoronary imaging (optical coherence tomography (OCT), intravascular ultrasound (IVUS) and invasive FFR (FFR))). The error bars indicate the 95% CIs.

Downstream secondary cardiovascular tests (excluding ICA) were selected from 92 different diagnostic codes (Supplementary Table 5). These cardiac imaging modalities were subcategorized into cardiovascular magnetic resonance imaging (MRI), cardiac CT, nuclear medicine, stress echocardiography and invasive intracoronary imaging (optical coherence tomography, intravascular ultrasound and invasive FFR). A total of 15,942 subsequent non-ICA cardiovascular tests were performed within 2 years of the index CCTA (178.5/1,000 patients). Noninvasive cardiovascular tests performed were lower at 2 years after FFR-CT (*n* = 6,777 (189/1,000 patients) versus 9,169 (167/1,000), *P* < 0.001) with a 12% relative risk reduction in the FFR-CT available cohort (HR 0.88 (0.85–0.92), *P* < 0.001) of having a downstream test. There was reduced likelihood of having a repeat cardiac CT (HR 0.87 (0.80–0.93), *P* < 0.001), second-line stress echocardiogram (HR 0.52 (0.44–0.62), *P* < 0.001) or nuclear stress testing (HR 0.61 (0.56–0.67), *P* < 0.001). The number of cardiovascular magnetic resonance scans performed was higher in the FFR-CT available cohort (HR 1.06 (1.00–1.13), *P* = 0.042). Invasive intracoronary imaging represented a small number of second-line tests (4.6/1,000 patients), but these increased significantly (HR 1.70 (1.36–2.12), *P* < 0.001) after FFR-CT availability (Fig. 3).

### Coronary revascularization

**Extended Data Fig. 6:**
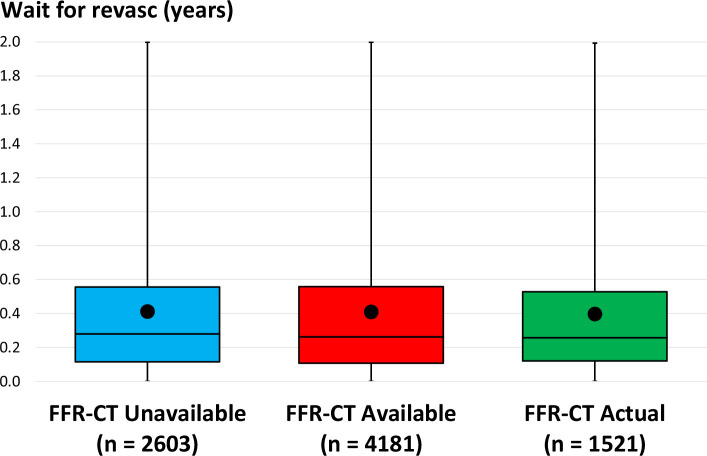
Referral to treatment time. Time (in years) from CCTA date (Time 0) to the date of revascularisation (PCI or CABG) for the 6,784 patients who had coronary revascularisation within the 2-year follow-up. The mean wait was 0.41 years for both FFR-CT unavailable and FFR-CT available groups. The group who received FFR-CT had a insignificant shorter wait (0.4 years). The box represents the interquartile range with the median as the middle segment, the mean as the black dot and the whiskers as minimum and maximum waits.

There was an early increase in PCI that was sustained at 2 years in the FFR-CT available group (*n* = 1,912 (5.4%) versus 3,161 (5.8%), aHR 1.09 (1.03–1.15, *P* = 0.002) (Table 2 and Supplementary Fig. 3). The likelihood of receiving coronary artery bypass grafting (CABG) did not change (*n* = 691 (1.9%) versus 1,020 (1.9%), aHR 1.01 (0.91–1.11), *P* = 0.89). Total revascularization rates (PCI and CABG) were higher in the FFR-CT available group (*n* = 2,603 (7.3%) versus 4,181 (7.6%), aHR 1.06 (1.01–1.11), *P* = 0.02). The proportion of patients going to ICA who received revascularization (revascularization ratio) was higher in the FFR-CT available cohort (48.3% versus 52.9%, *P* < 0.001) (Table 2). There was no significant difference in the treatment time from CCTA to revascularization between the FFR-CT unavailable and FFR-CT available cohorts nor the group who had adjunct FFR-CT analysis (Extended Data Fig. 6).

### FFR-CT subgroup analysis

Among the 7,863 patients who received FFR-CT analysis, 7,091 (90.1%) had a stenosis-specific result and 7,844 (99.7%) had a distal vessel result, leaving 19 (0.2%) with no FFR-CT value. A stenosis-specific positive FFR-CT (≤0.80) was observed in 4,390 (55.8%) patients. A positive FFR-CT predicted cardiovascular mortality (HR 3.00 (1.33–6.76), *P* = 0.008), MI (HR 4.76 (2.91–7.77), *P* < 0.001) and revascularization (HR 13.47 (10.74–16.89), *P* < 0.001) at 2 years. ICA rates after a positive FFR-CT were higher than for those with a negative FFR-CT, or no stenosis-specific FFR-CT value (FFR-CT ≤0.80, *n* = 2,406 (54.9%), FFR-CT >0.80, *n* = 299 (11.1%), no FFR-CT value, *n* = 36 (4.8%), *P* < 0.001) (Table 3).

**Table 3:** Event rates of the primary clinical outcomes for the FFR-CT tested group, with the risk of an event according to the FFR-CT result

	Stenosis pin >0.80 (*n* = 2,701)	Stenosis pin ≤0.80 (*n* = 5390)	Unadjusted HR (95% CI)	*P* value	No stenosis pin (*n* = 753)
All-cause mortality	56 (2.1)	115 (2.6)	1.27 (0.92–1.74)	0.15	10 (1.3)
Cardiovascular mortality	7 (0.3)	34 (0.8)	3.00 (1.33–6.76)	0.008	2 (0.3)
MI	18 (0.7)	137 (3.1)	4.76 (2.91–7.77)	<0.001	3 (0.4)
Revascularization (PCI or CABG)	79 (2.9)	1,439 (32.8)	13.47 (10.74–16.89)	<0.001	4 (0.5)
PCI	68 (2.5)	1,128 (25.7)	11.74 (9.19–15.00)	<0.001	4 (0.5)
CABG	11 (0.4)	311 (7.1)	18.02 (9.88–32.88)	<0.001	0 (0)
All ICA performed	299 (11.1)	2,406 (54.9)	6.86 (6.08–7.74)	<0.001	36 (4.8)
ICA with revascularization	79 (2.9)	1,439 (32.8)	13.48 (10.75–16.91)	<0.001	4 (0.5)
ICA no revascularization	220 (8.2)	967 (22.1)	2.95 (2.55–3.41)	<0.001	32 (4.2)

Cox-proportional hazard analysis for the primary outcomes at 2 years after index CT scan for the FFR-CT population (*n* = 7,863) using FFR-CT stenosis-specific values to categorize as positive (FFR-CT ≤0.80), negative (FFR-CT >0.80) or no FFR-CT value, with FFR-CT >0.80 as the reference comparator with unadjusted HR.

### AI implementation and learning

From a baseline in which no NHS site was commissioned in March 2018, within 12 months, 27 different hospitals implemented the AI technology. At the end of the program, 54 sites were commissioned and utilizing the AI technology in routine healthcare settings. National implementation was geographically equitable with balanced representation from across England, urban and rural, academic and nonacademic centers (Extended Data Fig. 1). The median time from funding to starting an FFR-CT program was 4.7 months (IQR 2.4–7.6) with some variability between centers and regions (North 4.6 (2.3–8.3), Midlands 0.8 (0.0–8.0), Southeast 5.4 (3.5–11.0), Southwest 5.1 (2.0–8.6)) (Extended Data Fig. 2b). The indices of multiple deprivation (IMD) score, which ranks populations from the least deprived to the most deprived areas, varied between hospitals according to geographic region and population served. There was no difference in the population IMD score before or after FFR-CT introduction (mean 20.58 (±15.9), pre-FFR-CT 20.61, post-FFR-CT 20.52), nor in the FFR-CT tested cohort (20.1), indicating that the availability and use of FFR-CT was nondiscriminatory according to the level of deprivation or social class (Supplementary Table 6).

The learning curve was determined on an institutional basis by assessing the patient outcomes and resource utilization from the FFR-CT tested cohort (*n* = 7,863) by the center experience (first 75 FFR-CT cases versus >75 cases). This demonstrated a change in practice and learning over time. The frequency of positive FFR-CT results increased (55% versus 60%), with no change in ICA (24.2% versus 23.8%) or revascularization (19.4% versus 19.4%) rates, resulting in an increased revascularization ratio (53.4% versus 56.2%). There was an associated reduction in the number of second-line downstream tests performed over time (220.5 versus 183.2/1,000 patients) (Supplementary Table 7).

## Discussion

This study has shown that a national technology implementation program enabled the rapid, equitable uptake of a new AI health technology in the health system. The introduction of FFR-CT was beneficial to the patient care pathway by reducing ICA and noninvasive functional cardiovascular tests performed 2 years after CCTA. The AI decision support tool appears safe with no significant difference in all-cause or CV mortality. The potential for increased risk of nonfatal MI during the technology availability will require longer-term assessment and consideration. AI implementation and clinical utility was balanced across populations, geographic location and institutional settings, with evidence of physician learning as use of the technology increased.

AI clinical decision support systems such as FFR-CT are being welcomed by overburdened healthcare systems with the aim of improving efficiencies and costs. Patients and clinicians often have greater apprehension about the use of AI in healthcare^14^. The introduction of new technologies and AI tools such as this constitute a complex health intervention in clinical practice and public health, aimed at improving patient outcomes. Assessing the real-world impact and clinical performance is difficult due to multiple interactions that may occur after a single health intervention^15^. These include differing groups or organizations being targeted, implementation of the AI technology outside of guidelines, and human factors such as the effects of time and learning^16^. Effectiveness, as assessed in randomized trials against usual treatment, frequently does not apply to real-world practice, limiting the impact and interpretability of trial results. Previous experience with computer-aided detection has highlighted the challenges in translation of trial results with use of technology versus real-world usage. In breast screening, for instance, the improved diagnostic accuracy in randomized controlled trials actually had reduced specificity with no benefit in the real world^17,18^. This study was designed as a real-world study to evaluate the national implementation of FFR-CT on patients and the wider health system by including every patient that underwent a CCTA at 27 different hospitals over a 3-year period, during which the technology was made available^19^.

This study provides insights into contemporary clinical practice and the type of patient undergoing investigation for suspected CAD^20^. A primary reason for NHS England recommending the adoption of FFR-CT was to improve the patient pathway by reducing unnecessary future downstream tests, especially invasive angiography. The clinical effectiveness of FFR-CT has previously been demonstrated by randomized trials showing lower rates of ICA compared with the usual treatment of noninvasive stress imaging tests at 9 and 12 months (refs. ^9,8^). Our real-world study population is 45 times larger than the comparator studies, has a longer follow-up and is more representative of contemporary clinical practice, as both cohorts were investigated with CCTA as the primary test under the same national guidance. After FFR-CT implementation, a reduction in all ICA and ICA without revascularization occurred early (90 days) and was sustained at 2 years after CCTA. National ICA rates were dropping before the introduction of FFR-CT related to the increased use of CCTA alone^21^, yet we have demonstrated that adding FFR-CT to this pathway further reduces rates of all coronary angiography by 7% and fewer inappropriate ICA procedures that did not result in treatment by 16%. The rate of ICA after a positive FFR-CT was a modest 55%. This points to the appropriate use of FFR-CT as an AI-diagnostic aid tool, where the AI-reported value gives a likelihood of functional significance (Extended Data Fig. 8), which must be integrated with the other imaging and clinical factors such as vessel size, location of the disease, or the patient being rendered asymptomatic on medications. The AI tool intends to assist clinicians and does not remove the need for clinical judgment.

Noninvasive downstream cardiovascular tests also reduced by 12% after the introduction FFR-CT, in particular the likelihood of having a repeat CCTA or nuclear stress test. A small but significant increase in the rates of cardiac MRI and invasive coronary tests were also observed. The increase in cardiac MRI despite greater use of CCTA after NICE guidance has been previously reported and does not appear to have been dampened by the first-line use of CCTA nor the addition of FFR-CT. An increase in invasive physiological and intracoronary imaging has also been observed but in this instance may reflect greater awareness and need to test stenoses after a positive FFR-CT, which previously would have been deemed unlikely to be flow limiting. This could be interpreted as improved clinical practice.

If FFR-CT is considered as another diagnostic test, the total number of diagnostics performed at 2 years is higher in the FFR-CT available group. However, from the patients’ perspective, FFR-CT is not another test, as it is a post-test AI analysis that occurs on the already acquired CCTA, saving further diagnostics referral waiting time, additional days missed from work and transport costs for hospital visits. The healthcare system could potentially also benefit from a reduction in overall test demand, which requires infrastructure (echo, MRI and CT equipment) and has direct workforce implications^5^. These are key paybacks that healthcare funders want to realize from the introduction of AI diagnostic aids.

Revascularization rates were higher after the FFR-CT available time period, with a small increase in PCI and a modest nonsignificant reduction in CABG. This shift of patients from CABG to PCI is consistent with the randomized controlled PRECISE study that compared a precision FFR-CT strategy with a routine cardiac testing strategy. This could reflect a change from three-vessel CAD, which is normally treated by CABG, to two- or one-vessel CAD, where PCI is more appropriate and feasible^22^. The impact of reduced ICA and increased PCI resulted in an improved revascularization ratio (patients at ICA receiving revascularization). This conversion rate of diagnostic test to treatment in one procedure is an important measure of FFR-CT efficacy compared with alternative non-AI, functional stress tests, which are associated with lower revascularization rates, thus wasting limited, expensive healthcare resources and putting the patient through an unnecessary invasive procedure with a risk of costly complications^23^.

**Extended Data Fig. 7:**
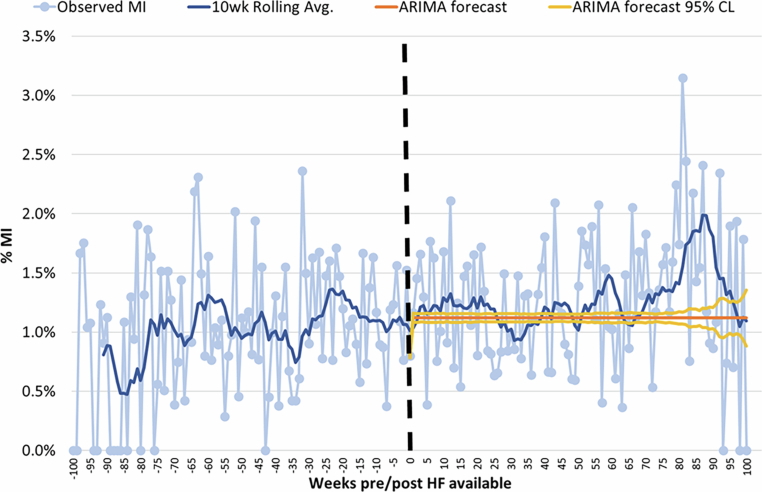
Autoregressive Integrated Moving Average curve for the incidence of myocardial infarction over teh study time period. Autoregressive Integrated Moving Average (ARIMA) method were performed. Weekly observed rates of all primary outcomes were assessed before and after FFR-CT introduction, then modelled for observed versus expected changes in outcomes post-health intervention over time. The dashed black line represents the time that FFR-CT was made available to the hospital sites.

**Extended Data Fig. 8:**
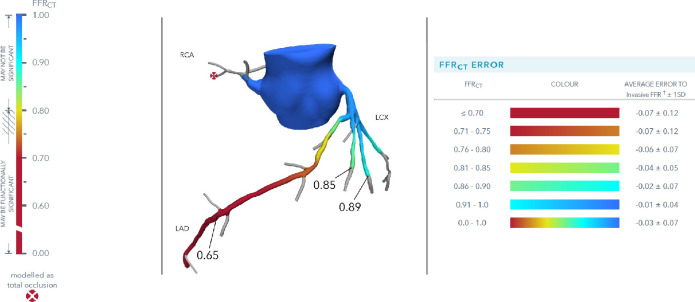
FFR-CT report example. A positive FFR-CT (centre panel). The LAD shows a gradual reduction from proximal to distal vessel with a stenosis specific value of 0.72 and distal vessel value of 0.65. The circumflex was ‘negative’ at 0.89 and the RCA was occluded. The scale on the left panel shows the degree of flow limitation and likelihood of functional significance as a continuum with the margins of error on the right panel to help the physician in their decision process.

Reduced unnecessary tests and improved CAD pathway efficiency are important to patients and healthcare providers, but any health intervention or new AI technology must first and foremost be safe^11,15^. We observed lower rates of all-cause mortality and cardiovascular mortality after the introduction of FFR-CT. The cohorts were overall well matched, with the group investigated before FFR-CT availability having a clinically small but a statistically significant increased number of risk factors such as heart failure, hypertension and kidney disease, whereas the post-FFR-CT group was older. These small differences in population risk factors probably explain the unadjusted lower all-cause and cardiovascular mortality rates observed in the FFR-CT available group, as their CAD burden should be similar given the knowledge that this does not change at a population level^24,25^. Higher PCI rates are unlikely to have had a significant impact, given that increased PCI has not been shown from studies in patients with stable CAD to improve mortality^26–30^. Overall, adjusted mortality and CV mortality were not different between groups and time periods, which is notable given the context of an adverse change in CV mortality trends since 2020 (ref. ^31^). The increased adjusted risk of MI in the FFR-CT available group at 2 years is difficult to interpret, especially given the lack of difference in unadjusted rates and on propensity matching (Extended Data Fig. 5). Increased MI events were observed in the PRECISE study and attributed to increased periprocedural events^8,32^. Our data show that the MI events increased in the FFR-CT available period with the event lines crossing at 90 days, similar to the increase in PCI (Extended Data Fig. 3). The FFR-CT available group had longer exposure to the coronavirus disease 2019 (COVID-19) pandemic period. This time period was associated with increased MIs and mortality in the general population and may be another possible explanation for differences observed between the groups, as MI events were higher late in the follow-up period that crossed over into the pandemic (Extended Data Fig. 7)^33,34^. It is difficult to attribute the increased adjusted risk of MI to the use of FFR-CT in 8.5% of the population, as there are many factors at play, including patient baseline risks, human factors and the pandemic.

One argument for using traditional functional stress tests over FFR-CT is the wealth of prognostic data available from them that is lacking in the FFR-CT literature. Small observational studies have suggested that a positive FFR-CT was associated with increased risk of MI and cardiovascular death compared with a negative FFR-CT; however, this was based on low numbers of events^35,36^. Due to the population size and number of clinical events in our study, we were able to show that FFR-CT provides patient-specific prognostic information, as the risk of MI or cardiovascular death increased three- to fivefold with a positive FFR-CT.

Our study suggests the potential of a centralized healthcare intervention program introducing AI technologies to improve AI utilization and support the transition to AI healthcare^15,37^. AI system challenges still exist, as, despite the technology being free to use, it took many centers up to 36 months to set up the information technology infrastructure and information governance processes required to use this cloud-based technology. Although the growth was substantial, in 3 years only 54 of 124 (44%) acute NHS England trusts were using the AI solution, despite unlimited central funding. High costs of FFR-CT would undoubtedly impact the affordability and, thus, accessibility for future broader use of this AI tool. Once implemented, the utilization of this AI technology was beneficial with generalizability across sites and populations (geography, age, gender and social deprivation). Human factors such as continuous learning appear to occur, with greater case selection and a reduction in additional testing associated with higher FFR-CT practice, thus implying that, with greater user experience, use of the technology can be adapted and potentially improve.

These results establish that the national introduction of a new AI technology FFR-CT by NHS England into the health system was beneficial to the patient chest pain pathway, supporting NICE’s recommendation and NHS England policymakers’ decision to centrally fund a new AI health technology. Implications for clinical practice could be wider utilization of FFR-CT to reduce unnecessary downstream tests. Future research will need to focus on the impact of increased revascularization, MI risk, longer-term impact and overall costs. Cost-effectiveness was fundamental to NICE’s recommendation and will be as important to the healthcare system as the clinical effectiveness of this health intervention^38^. There should be further investigation into the ability of FFR-CT to predict adverse cardiac events and whether it can be used to improve patient outcomes by more personalized patient-specific decision-making and revascularization.

Strengths of this study are its quasi-experimental observational design that resulted in total population inclusion, thus reducing any selection bias. The use of hospital-linked outcomes data reduced recollection bias that can be a limitation of observational studies, and it is reassuring that many of the key findings of this study are consistent with the randomized trial evidence^9^. There remains the risk of information bias utilizing HRs to adjust for imbalances in baseline potential prognostic confounders; thus, we supplemented our analysis with a PSM model.

The study has several weaknesses. As an observational study, causation cannot be attributed but should be considered in the context of wider multifactorial confounders. The use of routinely collected healthcare data enables large-scale studies such as this and limits misclassification, observer and recall bias but may be imperfect in the lack of adjudication of every event. The data are based on clinical coding and International Classification of Diseases (ICD) subgroups and so, on occasion, lack granularity, such as the inability to differentiate the type of MI. Patients were followed up for 2 years at different time points, rather than a single longitudinal cohort design. Cohort assignment depended upon when the patient had their scan and whether the AI technology was available at their hospital. Thus, referral bias cannot be totally excluded, as changes in practice can occur over time regardless of guidelines. The benefit of utilizing FFR-CT in the English NHS cannot be generalized to all healthcare systems, as each health intervention is unique to the environment in which it is implemented. The COVID-19 pandemic occurred during the study, meaning that the post-FFR-CT group had a greater follow-up time period during COVID. Our analysis suggests this had no significant impact on downstream test differences.

In conclusion, this study shows that a widescale national implementation of an AI diagnostic aid was feasible and resulted in widespread use of the technology. The use of FFR-CT in the stable CAD pathway was beneficial by reducing subsequent invasive and noninvasive cardiac tests and safe with no difference in MIs, all-cause or CV mortality. Implications for clinical practice could be the wider utilization of FFR-CT to reduce unnecessary downstream tests, and health policymakers may advocate more directed adoption programs to increase the uptake of AI tools within the health system.

## Methods

### Study design and population

FISH&CHIPS was a multicenter, retrospective, quasi-experimental, observational cohort study. The study aimed to determine the safety and benefit of FFR-CT introduction into NHS England by comparing a clinical population undergoing CCTA for investigation of possible stable CAD after the introduction of FFR-CT with a previous ‘standard of care’ population undergoing CCTA before FFR-CT availability with the use of existing second-line functional cardiac investigations (such as myocardial perfusion scintigraphy, stress echocardiography and perfusion cardiac MRI).

Hospitals participating in the NHS Innovation Technology Payment (ITP) program were invited. Sites were required to have performed >50 FFR-CT studies in the first year of their program to reduce learning curve bias. Participants were required to be age ≥18 years and have undergone a CCTA from April 2017 to December 2020. Nondedicated CCTA scans including CT calcium score alone, CT aorta and CT for transcatheter aortic valve implantation planning were excluded.

The study population was divided into two groups based on whether their CCTA was performed before or after FFR-CT became available at their hospital. All patients were investigated under the same NICE chest pain guidelines, with total inclusion; thus, both groups should be comparable and representative of a typical chest pain population, without the preselection bias often found in randomized controlled trials.

### Data source

Clinical events were determined from routinely collected healthcare data from April 2016 to April 2022, using a previously validated methodology^39^. This provided 6 years of event data with a median 1 year prior and 3.3 years follow-up after CCTA. Data were requested before CCTA to ensure accurate baseline patient characterization. Data from all hospital episodes, including diagnostic coding, were obtained from NHS Digital’s Data Access Reporting System. This comprised the hospital episode statistics (HES) admitted patient care (APC), critical care (CC), emergency care (ECDS) and outpatient care (OPC) datasets. Diagnostic tests performed were captured in the diagnostic imaging dataset (DIDS). Mortality data and cause of death were obtained from the Office for National Statistics (ONS)-linked HES dataset. FFR-CT analysis was performed by a commercial company (HeartFlow) in accordance with the ITP funding. The decision to send a CCTA for FFR-CT analysis was at the discretion of the clinical team. FFR-CT was performed on a per-patient and per-vessel basis, then reported to the physician for clinical interpretation. Pseudonymized FFR-CT analysis was provided by HeartFlow to the study team for data linkage. Analysis of the FFR-CT data is based on the lowest stenosis specific value (2 cm distal to a stenosis) reported per patient.

### Study outcomes

The study was designed to evaluate the early-stage use of an AI decision support system in accordance with reporting guidelines, by focusing on the clinical utility, safety and human factors associated with AI implementation^40^. Differences in health-related events between the populations were used to determine the primary objectives of safety and impact of implementing FFR-CT on the health system. Primary safety outcomes included all-cause mortality, cardiovascular mortality and MI event rates. Primary impact outcomes included the rates of downstream tests performed after the index CCTA; including ICA with and ICA without revascularization as well as noninvasive functional tests or repeat CCTA. Secondary analysis categorized FFR-CT patients’ outcomes according to their FFR-CT result. Human factors were assessed by describing the uptake and utilization across different sites and population demographics, as well as any impact of learning over time.

Clinical outcomes were reported using the hospital admission diagnostic codes from the OPCS Classification of Interventions and Procedures (OPCS-4) and equivalent International Statistical Classification of Diseases and Related Health Problems 10th Revision (ICD-10)-World Health Organization version definitions. Cardiovascular deaths included I00-I99, excluding I26-28 (pulmonary heart disease), I60-69 (cerebrovascular disease) and I80-89 (diseases of the veins and lymphatics). MI was defined as a new admitted patient episode with a diagnostic code of MI (I21/I22) (SupplementaryTable 4).

Downstream cardiovascular diagnostic tests were collected from the DIDS, categorized as per the National Interim Clinical Imaging Procedure code set used for the coding of clinical imaging procedures in electronic systems in the NHS.

### Statistical analysis

Descriptive data continuous variables are reported as mean (± standard deviation) or median (IQR) and categorical as number (%). Comparisons were performed using Student’s *t*-test, Mann–Whitney test or chi-square test where appropriate. Time to first event was calculated using Kaplan–Meier methodology from the time of the CCTA until 2 years. Cox-proportional regression was used to determine the univariable HR of clinical outcomes at 2 years after CCTA^41–43^. Prognostic covariates that were different between the groups at entry into the study were entered into an adjusted multivariable Cox regression model^41,44^. Additional multivariable sensitivity analysis were performed using the differences in baseline characteristics with the inclusion of FFR-CT availability as an interaction term for predicting ICA. Age was recalculated as mean-centered age for the interaction term to reduce multicollinearity. PSM was performed for the primary outcomes to help determine causal effect. The PSM model used a 1:1 nearest-neighbor method, with assessment of post-match covariate by standardized mean difference. A standardized mean difference of <5% was used for the matching. Matched-pairs analysis was performed using Cox-proportional hazard models stratified on the matched pairs. The 95% confidence intervals (CIs) are reported with *P* values, and a two-sided *P* value <0.05 was considered statistically significant. All statistical analysis was performed in the R stats package (R documentation, version 3.6.2).

### Ethics and consent

The Confidentiality Advisory Group (CAG) approved the use of confidential patient information without consent on the basis of health and social care research in the public interest (National Health Service Act 2006, s251, ‘Control of patient information’; CAG reference 20CAG0101). Ethical approval was obtained from the Health Regulatory Authority (Integrated Research Application System project ID 285996; Research Ethics Committee reference 20/NW/0430). The NHS ‘Opt out of research’ database was queried with participants excluded if consent was withdrawn. The study was performed in accordance with the Declaration of Helsinki and principles of good clinical practice.

### Reporting summary

Further information on research design is available in the Nature Portfolio Reporting Summary linked to this article.

## Online content

Any methods, additional references, Nature Portfolio reporting summaries, source data, extended data, supplementary information, acknowledgements, peer review information; details of author contributions and competing interests; and statements of data and code availability are available at 10.1038/s41591-025-03620-y.

## Supplementary information

Supplementary Information Methods, Supplementary Tables 1–7, Stastical analysis plan and Protocol.

Reporting Summary

## Data Availability

Supporting data are available via the UK Data service repository at https://reshare.ukdataservice.ac.uk and via figshare at 10.6084/m9.figshare.28398374 (ref. ^45^), including the study protocol and the algorithms for defining clinical outcomes from HES data. Dataset availability is subject to controlled access due to the CAG approvals and NHS England’s data sharing contract (CON-317153-H1H4Z (version 2.03)). Individual deidentified, aggregated participant data that underlie the study reported outcomes will be made available in accordance with the ethical approvals, NHSE data sharing framework contract and the Medical Research Council Industrial Collaboration Agreement (MICA). Any data sharing is subject to a data sharing agreement (DSA) between parties. Each DSA will detail: (1) the data to be provided; (2) the legal basis for sharing data; (3) the purpose of the sharing and use of the data; (4) the expected benefits to health and/or social care by sharing the data; (5) the data transfer method; (6) any associated DSAs; (7) any special terms and conditions for the use or reuse of the data; and (8) any charges payable for the provision of the data. Requests for data sharing should be communicated in writing to the research governance team at Liverpool Heart and Chest Hospital (Research.Governance@lhch.nhs.uk) specifying the nature of the request. All external requests will be responded to within 2 weeks by the sponsors director of research, with an estimated timeframe of 3 months from the date of request to DSA approval. The study ethics approvals allow data storage up to 15 years.

## Code availability

The code used to determine clinical events from the HES datasets is available via the UK Data service repository at https://reshare.ukdataservice.ac.uk; search ‘Implementation of a national AI technology program on cardiovascular outcomes and the health system’ (10.5255/UKDA-SN-857706). The underlying methodology for FFR-CT computational analysis has been published previously; however, the source code for FFR-CT computation is proprietary, so it cannot be made available publicly.
