# Supplementary material for: Implementation of a national AI technology program on cardiovascular outcomes and the health system
Source: Nat Med. 2025 Apr 4;31(6):1903–10. doi: 10.1038/s41591-025-03620-y (PMC12176617; doi:10.1038/s41591-025-03620-y)
Supplement: Supplementary file 1 — Methods, Supplementary Tables 1–7, Stastical analysis plan and Protocol. [file 41591_2025_3620_MOESM1_ESM.pdf]

nature medicine

Supplementary information https://doi.org/10.1038/s41591-025-03620-y

Implementation of a national AI technology program on cardiovascular outcomes and the health system

In the format provided by the authors and unedited

Supplementary Material

Supplementary Table 1

Patient characteristics of the FFR-CT tested population at baseline

	FFR-CT Subgroup n (%) 7863 (8.7)
**Demographics**	
Age at index CT scan (years)	63 (55, 71)
Female	3023 (38.7)
Type 1 Diabetes Mellitus	114 (1.5)
Type 2 Diabetes Mellitus	1224 (15.6)
Hyperlipidaemia	2627 (33.5)
Hypertension	3918 (50.0)
Angina	2454 (31.3)
Myocardial Infarction	325 (4.1)
Valve Disorder	618 (7.1)
Heart Failure	681 (8.7)
TIA	73 (0.9)
Cerebral Infarction	133 (1.7)
Atherosclerosis	121 (1.5)
Aortic Aneurysm	190 (2.4)
COPD	911 (11.5)
Kidney Disease	516 (6.6)

Data are presented as number and %
or number and 95th Confidence Interval

Supplementary Table 2 Multivariable sensitivity analysis with FFRCT availability as an interaction term for predicting ICA

.

	coef	se	p-value	coef	se	p-value	coef	se	p-value	coef	se	p-value
FFR-CT available	-0.052	0.019	0.007	-0.066	0.034	0.052	-0.052	0.020	0.009	-0.059	0.019	0.002
Age at index CT scan (years)*	0.017	0.001	<0.001	0.017	0.001	<0.001	0.017	0.001	<0.001	0.017	0.001	<0.001
Heart failure	0.075	0.026	0.004	0.075	0.026	0.005	0.051	0.037	0.17	0.075	0.026	0.005
Hypertension	1.230	0.021	<0.001	1.212	0.032	<0.001	1.230	0.021	<0.001	1.230	0.021	<0.001
Valve disease	0.302	0.024	<0.001	0.301	0.024	<0.001	0.301	0.024	<0.001	0.302	0.024	<0.001
COPD	0.072	0.027	0.008	0.072	0.027	0.008	0.072	0.027	0.008	0.072	0.027	0.008
Kidney disease	-0.067	0.029	0.02	-0.067	0.029	0.02	-0.067	0.029	0.02	-0.140	0.044	0.001
FFR-CT available * Mean-centred age	0.002	0.001	0.27									
FFR-CT available * Hypertension				0.03	0.04	0.45						
FFR-CT available * Heart failure							0.042	0.046	0.36			
FFR-CT available * Kidney disease										0.127	0.055	0.02

* Age was recalculated as mean-centered age for the interaction term to reduce multicollinearity.

Supplementary Table 3 Propensity Matched Demographics and co-morbidities with the standardized mean difference (SMD)

	FFR-CT unavailable (n = 30665)	FFR-CT available (n = 30665)	SMD
Age at index CT scan (years)	57 (49 to 66)	57 (49 to 66)	-0.50
Female	15702 (51.2)	16009 (52.2)	2.00
Type 1 Diabetes mellitus	183 (0.6)	141 (0.5)	-1.21
Type 2 Diabetes mellitus	3301 (10.8)	3465 (11.3)	1.55
Hyperlipidaemia	6110 (19.9)	6201 (20.2)	0.70
Hypertension	11252 (36.7)	11269 (36.8)	0.11
Angina	4583 (15)	4673 (15.2)	0.76
Myocardial infarction	746 (2.4)	697 (2.3)	-0.78
Valve disorder	2483 (8.1)	2402 (7.8)	-0.81
Heart failure	1948 (6.4)	1930 (6.3)	-0.20
TIA	103 (0.3)	101 (0.3)	-0.07
Cerebral infarction	267 (0.9)	298 (1)	0.73
Atherosclerosis	175 (0.6)	179 (0.6)	0.11
Aortic aneurysm	370 (1.2)	372 (1.2)	0.04
COPD	1721 (5.6)	1713 (5.6)	-0.10
Kidney disease	1187 (3.9)	1192 (3.9)	0.07

Supplementary Table 4

Hospital Admission diagnostic codes from the OPCS Classification of Interventions and Procedures (OPCS-4) and equivalent International Statistical Classification of Diseases and Related Health Problems 10th Revision (ICD-10)-WHO Version for cause of death

	**Hospital admission codes**	**Mortality codes**
**All Cause death**		**A00-Z99**
**Cardiovascular death**	**I00-I99**	**I100-102** Acute rheumatic fever<br>**I105-109** Chronic rheumatic heart diseases<br>**I10-I15** Hypertensive diseases<br>**I20-I25** Ischaemic heart diseases<br>**I30-I52** Other forms of heart disease<br>**I70-I79** Diseases of arteries, arterioles and capillaries<br>**I95-I99** Other and unspecified disorders of the circulatory system
**Ischaemic Heart Disease**	**I20-I25**	**I21** Acute Myocardial Infarction<br>**I22** Subsequent myocardial infarction<br>**I23** Certain current complications following acute myocardial infarction<br>**I24** Other acute ischaemic heart diseases

		**I25** Chronic ischaemic heart disease
**Myocardial Infarct**	**I21-22**	**I21** Acute myocardial infarction<br><br>**I22** Subsequent myocardial infarction
**Cerebrovascular accident**	**I60-I69**	**I60** Subarachnoid haemorrhage<br><br>**I61** Intracerebral haemorrhage<br><br>**I62** Other nontraumatic intracranial haemorrhage<br><br>**I63** Cerebral infarction<br><br>**I64** Stroke, not specified as haemorrhage or infarction
**Invasive Coronary Angiogram**	**K63** Contrast radiology of heart<br><br>**K65.3** Catheterisation of left side of heart NEC	
**Percutaneous coronary intervention**	**K49** Percutaneous transluminal laser coronary angioplasty<br><br>**K50.1** Other therapeutic transluminal operations on coronary artery<br><br>**K75** Percutaneous transluminal balloon angioplasty and insertion of stent into coronary artery<br><br>**K76** Transluminal operations on cardiac conduit	
**Coronary artery bypass grafting**	**K40-46**	

Supplementary Table 5

The 92 Cardiovascular diagnostic tests acquired from the Diagnostic Imaging Dataset (DIDS), categorized as per the National Interim Clinical Imaging Procedure code set (NICIP) used for the coding of clinical imaging procedures in electronic systems in the NHS.

NCIP short code	NCIP Description	SCT-ID	SNOMED description
CACRY	CT Cardiac angiogram coronary	419545005	Computed tomography angiography of coronary arteries (procedure)
FACLV	Cardiac Angio Lt heart study	420136008	Fluoroscopic angiography of left side of heart (procedure)
FACRH	Cardiac Angio Rt heart study	419177004	Fluoroscopic angiography of right side of heart (procedure)
FCARD	Cardiac Angio coronaries	419416005	Fluoroscopic angiography of coronary arteries (procedure)
FHART	Fluoroscopy heart	82327001	Cardiac fluoroscopy (procedure)
IACOAS	Cardiac Angio coronary stent	418982001	Fluoroscopic angiography of coronary artery and insertion of stent (procedure)
MCARD	MRI Heart	241620005	Magnetic resonance imaging of heart (procedure)
MCORP	MRI Cardiac perfusion	419535008	Magnetic resonance imaging perfusion study of heart (procedure)
NCMPS	NM Cardiac myocardial perfusion scan	252432008	Radionuclide myocardial perfusion study (procedure)
CAAAG	CT Angiogram aorta	305053005	Computed tomography of aorta (procedure)
CCASC	CT Cardiac Cor artery calcium scoring	426005005	Cardiac computed tomography for calcium scoring (procedure)
FCARC	Cardiac Angio congenital anomaly study	322201000000102	Fluoroscopic percutaneous angiography of heart for congenital anomaly study (procedure)
UTOEG	US Transoesophageal echocardiogram	105376000	Transesophageal echocardiography (procedure)
IACOAP	Cardiac Angio PTCA	429809004	Fluoroscopic percutaneous transluminal angioplasty of coronary artery (procedure)
ICORD	Intracoronary doppler	431747006	Intravascular ultrasound doppler imaging of coronary artery using fluoroscopic guidance (procedure)
IPWCAI	Cardiac pressure wire and Cath insertion	433031009	Cardiac catheterization with insertion of pressure wire using fluoroscopic guidance (procedure)
IPWPCI	Cardiac pressure wire and PCI	431558000	Insertion of cardiac pressure wire using fluoroscopic guidance (procedure)

MAAOW	MRA Aorta whole	431431005	Magnetic resonance imaging angiography of whole aorta (procedure)
MCRPS	MRI Cardiac rest perfusion	431392001	Magnetic resonance imaging of rest perfusion of heart (procedure)
MCSFS	MRI Cardiac stress function study	431609005	Magnetic resonance imaging stress study of cardiac function (procedure)
MCSPS	MRI Cardiac stress perfusion	431299007	Magnetic resonance imaging of perfusion of heart under stress (procedure)
MCVFS	MRI Cardiac valvular function study	432845009	Magnetic resonance imaging of cardiac valvular function (procedure)
MCVIA	MRI Cardiac myocardial viability	431940000	Magnetic resonance imaging of heart for assessment of myocardial viability (procedure)
MCVVS	MRI Cardiac ventricular volume study	432846005	Magnetic resonance imaging of cardiac ventricular volume (procedure)
NCARDO	NM Cardiac rest PET FDG	432026001	Positron emission tomography myocardial rest imaging using fluorodeoxyglucose (procedure)
NCARGO	NM Cardiac rest gated PET FDG	434267000	Positron emission tomography electrocardiography gated myocardial rest study using fluorodeoxyglucose (procedure)
NCARSO	NM Cardiac stress PET Rb81	431901005	Positron emission tomography myocardial stress imaging using rubidium 81 (procedure)
NCARVO	NM Cardiac viability scan PET FDG	433227008	Positron emission tomography of heart for cardiac viability using fluorodeoxyglucose (procedure)
NCFPS	NM Cardiac first pass angiogram	431942008	Radionuclide cardiac first pass angiography (procedure)
NCGRTO	NM MPS Thallium rest gated	431644007	Radionuclide electrocardiography gated myocardial perfusion rest study using thallium 201 (procedure)
NCNRGO	NM Cardiac rest gated PET NH3	432943001	Positron emission tomography electrocardiography gated myocardial rest study using N13 ammonia (procedure)
NCRRNO	NM Cardiac rest PET Rb81	432847001	Positron emission tomography myocardial rest imaging using rubidium 81 (procedure)
NMTLSO	NM MPS Thallium stress	431511008	Myocardial perfusion stress imaging using Thallium 201 (procedure)
NMTSGO	NM MPS Thallium stress gated	433630009	Radionuclide electrocardiography gated myocardial perfusion stress study using thallium 201 (procedure)

NRVEN	NM Cardiac ventriculography rest	432115009	Radionuclide ventriculography at cardiac rest (procedure)
NRVENO	NM Cardiac ventriculography rest SPECT	429801001	Single photon emission computed tomography ventriculography at cardiac rest (procedure)
NSRNVO	NM Cardiac ventriculography stress SPECT	429821000	Single photon emission computed tomography cardiac stress ventriculography (procedure)
NSVEN	NM Cardiac ventriculography stress	432155006	Radionuclide ventriculography at cardiac stress (procedure)
UTE3D	US Transthoracic echocardiogram 3D	434158009	Transthoracic three dimensional ultrasonography of heart (procedure)
UTECGC	US TTE with Contrast	434167009	Transthoracic ultrasonography of heart with contrast (procedure)
UTESDC	US Stress echocardiogram with contrast	433862009	Exercise stress ultrasonography of heart with contrast (procedure)
UTIES	US Tissue strain rate echocardiogram	431343007	Ultrasonography of myocardium for tissue strain rate (procedure)
IPAVR	Percutaneous aortic valve replacement	377151000000105	Percutaneous aortic valve replacement using fluoroscopic guidance (procedure)
UTOEGC	US TOE with contrast	440467009	Transesophageal echocardiography with contrast (procedure)
NCCAV	NM Cardiac viability scan FDG PET CT	443249000	Positron emission tomography with computed tomography of heart for cardiac viability using fluorodeoxyglucose (procedure)
NCCAG	NM Cardiac rest gated FDG PET CT	443300009	Positron emission tomography using fluorodeoxyglucose with computed tomography electrocardiography gated myocardial rest study (procedure)
NCCNG	NM Cardiac rest gated N13 PET CT	443277009	Positron emission tomography using nitrogen 13 ammonia with computed tomography electrocardiography gated myocardial rest study (procedure)
NCCRF	NM Cardiac rest FDG PET CT	443534009	Positron emission tomography using fluorodeoxyglucose with computed tomography myocardial rest imaging (procedure)
NCCRE	NM Cardiac rest Rb81 PET CT	443628005	Positron emission tomography using rubidium 81 with computed tomography myocardial rest imaging (procedure)

NCCRN	NM Cardiac rest N13 PET CT	443535005	Positron emission tomography using nitrogen 13 ammonia with computed tomography myocardial rest study (procedure)
NCCAS	NM Cardiac stress Rb81 PET CT	443629002	Positron emission tomography using rubidium 81 with computed tomography myocardial stress imaging (procedure)
NMPSM	NM MPS MIBI rest	252434009	Cardiac Tc-99m methoxyisobutyl isonitrile study (procedure)
ITAAVI	Pc transapical insertion aortic valve	444614006	Percutaneous transapical insertion of aortic valve using fluoroscopic guidance (procedure)
CAOWHC	CT Aorta whole with contrast	444969006	Computed tomography of entire aorta with contrast (procedure)
CAOTHC	CT Aorta thoracic with contrast	444970007	Computed tomography of thoracic aorta with contrast (procedure)
CCAGA	CT Cardiac gated	241547009	Computed tomography of heart (procedure)
CCAGFC	CT Cardiac gated function with contrast	772801000000101	Gated computed tomography for cardiac function with contrast (procedure)
CCGCCC	CT Cardiac gated complex congenital Cont	772821000000105	Gated computed tomography for complex congenital heart disease with contrast (procedure)
UTO3D	US Transoesophageal echocardiogram 3D	445864005	Three dimensional transesophageal ultrasonography of heart (procedure)
NMMRG	NM MPS MIBI rest gated	446182004	Radionuclide electrocardiography gated myocardial perfusion rest study using technetium Tc^99m^ methoxyisobutylisonitrile (procedure)
CCAGAC	CT Cardiac gated with contrast	448431001	Gated computed tomography of heart with contrast (procedure)
NCPRTO	NM MPS Thallium rest and redistribution	826691000000108	Radionuclide myocardial perfusion rest and redistribution study using thallium 201 (procedure)
NCTRGO	NM MPS Thallium rest and Redist gated	826711000000105	Radionuclide electrocardiography gated myocardial perfusion rest and redistribution study using thallium 201 (procedure)
NCPCTO	NM MPS Thallium stress and Redist	826741000000106	Radionuclide myocardial perfusion stress and redistribution study using thallium 201 (procedure)

NJCGCO	NM MPS Tl stress Redist and re-injection	440335005	Radionuclide imaging of perfusion of myocardium under stress and reinjection using Thallium 201 (procedure)
MCAMFC	MRI Card morphology Func with contrast	449879007	Magnetic resonance imaging for cardiac morphology and function with contrast (procedure)
MCHIL	MRI Cardiac and hepatic iron load	442086001	Magnetic resonance imaging of heart and liver for assessment of cardiac and hepatic iron load (procedure)
MCMFS	MRI Card morphology function stress	449882002	Magnetic resonance imaging for cardiac morphology and function under stress (procedure)
MCMFSC	MRI Card Morph Func stress with contrast	449883007	Magnetic resonance imaging for cardiac morphology and function under stress with contrast (procedure)
MCMFV	MRI Card morphology Func velocity stress	449878004	Magnetic resonance imaging for cardiac morphology, function, and velocity under stress (procedure)
MCMFVC	MRI Card Morph Func velocity with Cont	449880005	Magnetic resonance imaging for cardiac morphology, function, and velocity with contrast (procedure)
MCMOP	MRI Cardiac morphology function	449876000	Magnetic resonance imaging for cardiac morphology and function (procedure)
MCMPV	MRI Card morphology function velocity	449877009	Magnetic resonance imaging for cardiac morphology, function, and velocity (procedure)
MMFVSC	MRI Card Morph Func velocity stress Cont	449881009	Magnetic resonance imaging for cardiac morphology, function, and velocity under stress with contrast (procedure)
IPCI	Percutaneous coronary intervention	415070008	Percutaneous coronary intervention (procedure)
NSRB82	NM Cardiac stress PET Rb82	818291000000100	Positron emission tomography myocardial stress imaging using rubidium 82 (procedure)
NRRB82	NM Cardiac rest PET Rb82	818301000000101	Positron emission tomography myocardial rest imaging using rubidium 82 (procedure)
CCORGC	CT Angio coronary artery graft	450506009	Computed tomography angiography of coronary artery bypass graft (procedure)
MAORTC	MRI Aorta with contrast	450527004	Magnetic resonance imaging of aorta with contrast (procedure)
MAOTHC	MRI Aorta thoracic with contrast	450528009	Magnetic resonance imaging of thoracic aorta with contrast (procedure)
NCNRNO	NM Cardiac rest PET NH3	241441008	Positron emission tomography (procedure)
MACOA	MRA Cardiac coronary arteries	419997008	Magnetic resonance imaging (procedure)

CMUGA	CT Cardiac multiple gated acquisition	431190000	Computerized axial tomography (procedure)
UCORA	US Intracoronary	431945005	Diagnostic ultrasonography (procedure)
FALCG	Cardiac Angio LV and Coronary Graft	440332008	Fluoroscopy (procedure)
NMPTF	NM MPS Tf rest	446874001	Nuclear medicine procedure (procedure)
NMTFS	NM MPS Tf stress gated	446875000	Nuclear medicine procedure (procedure)
NMIST	Radionuclide myocardial perfusion stress study using technetium Tc^99m^ methoxyisobutylisonitrile	446876004	Nuclear medicine procedure (procedure)
NMTRG	NM MPS Tf rest gated	447525000	Nuclear medicine procedure (procedure)
NMSTT	NM MPS Tf stress	447526004	Nuclear medicine procedure (procedure)
NMMSG	Radionuclide electrocardiography gated myocardial perfusion stress study	447586001	Nuclear medicine procedure (procedure)
CTHXG	Gated CT thorax	858161000000109	Computerized axial tomography (procedure)
FOCAL	OCT Coronary artery Fluoro guided Left	912321000000101	Fluoroscopy (procedure)
FOCAR	OCT Coronary artery Fluoro guided Left	912321000000101	Fluoroscopy (procedure)

Supplementary Table 6: Socio-economic status of the overall population, per site and in those patients receiving the addition of the AI diagnostic decision tool

Hospital	IMD04	IMD pre-FFRCT	IMD post-FFRCT	IMD FFRCT tested	FFRCT utilisation (%)
ASP	10.06 (±6.2)	10.5	10.8	10.2	6.3
BGH	18.45 (±10.7)	17.5	18.2	18.8	8.4
BHL	26.58 (±19.4)	29.0	29.4	26.7	12.2
BRI	19.56 (±14.7)	21.8	21.5	20.8	9.6
BVH	19.65 (±15.2)	25.4	25.9	20.2	4.8
EXE	16.53(±8.8)	16.6	16.7	16.9	3.1
FPH	14.2 (±10.2)	15.4	15.3	15.2	8.4
GWH	12.69 (±8.6)	14.4	13.9	12.6	17.2
HEY	25.78 (±19.7)	24.8	25.7	25.0	8.4
HMN	22.62 (16.9)	23.5	21.9	21.0	3.5
LGI	25.95 (±18.9)	25.7	26.2	25.5	8.1
MFT	28.98 (±18.6)	29.4	29.8	27.0	16.6
NUH	27.42 (±19.1)	27.3	28.2	27.1	16.8
PTM	18.28 (±12.9)	18.8	18.3	18.2	1.1
RHX	24.93 (±14.6)	23.6	23.7	22.9	5.4
RPH	14.29 (±10.1)	15.2	15.6	14.4	7.3
RUH	13.24 (8.7)	13.9	13.0	12.7	20.8
STH	28.62 (±18.7)	27.2	27.7	28.9	7.2
UHD	15.15 (±10.8)	16.6	16.4	15.6	8.2
UHL	19.47 (±14.5)	20.8	20.4	19.4	5.7
UHS	21.27 (±14.8)	19.4	19.5	21.7	8.2
ULH	18.75 (±10.4)	18.8	20.1	17.9	11.4
UNT	29.46 (±19.1)	29.2	29.0	26.9	11.1
WHH	11.33 (±7.7)	12.4	12.5	11.3	10.9
WSH	15.63 ((±8.7)	15.7	15.5	15.3	8.5
**All sites**	20.58 ((±15.9)	20.52	20.61	20.1	8.7

Supplementary Table 7: Learning curve of using the AI decision support tool

	**Stenosis specific FFRCT ≤0.8* n(%)**		**Invasive Coronary Angiography n(%)**		**Revascularisation n(%)**		**Revascularisation ratio n(%)**		**Downstream Cardiac stress tests (rate per 1000 patients)**	
	**≤ 75 cases**	**> 75 cases**	**≤ 75 cases**	**> 75 cases**	**≤ 75 cases**	**> 75 cases**	**≤ 75 cases**	**> 75 cases**	**≤ 75 cases**	**> 75 cases**
**Patients**	951 / 1713 (55.5%)	3246 / 5371 (60.4%)	454 / 1873 (24.2%)	1420 / 5963 (23.8%)	364 / 1873 (19.4%)	1158 / 5963 (19.4%)	364 / 682 (53.4%)	1158 / 2059 (56.2%)	413 / 1873 (220.5)	1092 / 5963 (183.2)

*Stenosis specific (2cm distal to the stenosis) patients (n=7091) were used for clinical interpretation.

Extended Data Figures

Extended Data Figure 1

A map of NHS England with the 25 different NHS Trusts plotted, their corresponding Integrated Care Board (ICB) and number of patients (n) contributed to the study.

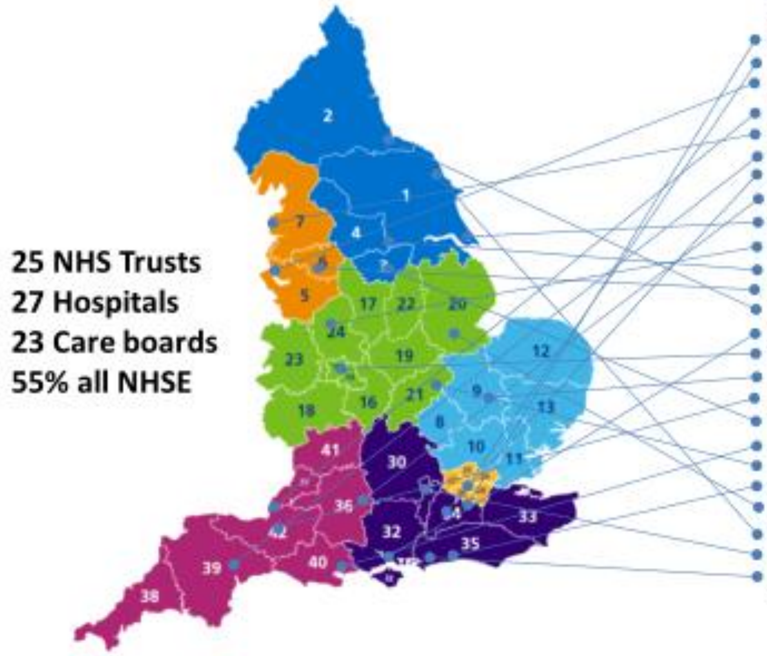

Site	Patients (n)
Ashford and St Peters	1272
Royal Brompton and Harefield	6708
Liverpool Heart and Chest	4477
Bristol Royal Infirmary	2997
Blackpool Victoria	2254
Royal Devon and Exeter	2192
Frimley Health	4347
Great Western Hospitals	2344
Hull and East Yorkshire	3068
University Hospitals North Midlands	4834
Leeds General Infirmary	2091
Manchester Unversity Trust	10136
Newcastle Upon Tyne Hospitals	3140
Portsmouth Hospitals	4196
Russells Hall Hospital	2905
Royal Papworth Hospital	6805
Royal United Hospitals Bath	2450
Sheffield Teaching Hospitals	2638
University Hospitals Dorset	4324
Univeristy Hospitals of Leicester	4484
University Hospitals Sussex	3785
United Lincolnshire Hospitals	1718
University Hospitals North Tees	1028
West Hertfordshire Hospital	4033
Western Sussex Hospitals	2347

Extended Data Figure 2

A. Patient recruitment and drop out for 6 months, 12 months and 24 month time points: Categorized as pre (blue line) or post (red line) FFR-CT availability. All 27 sites provided patient data from April 2017 – April 2020 with 2 sites (UHD and BRI) providing data from April 2017-December 2020.
B. Site FFR-CT availability: Introduction of FFR-CT at a site level was used to define whether the CCTA was performed before or after FFR-CT was made available at their site. The bubbles represent the time that FFR-CT was made available for clinical use and the number of CCTA scans performed at each site over the study recruitment time period (April 2017-December 2020).

A.

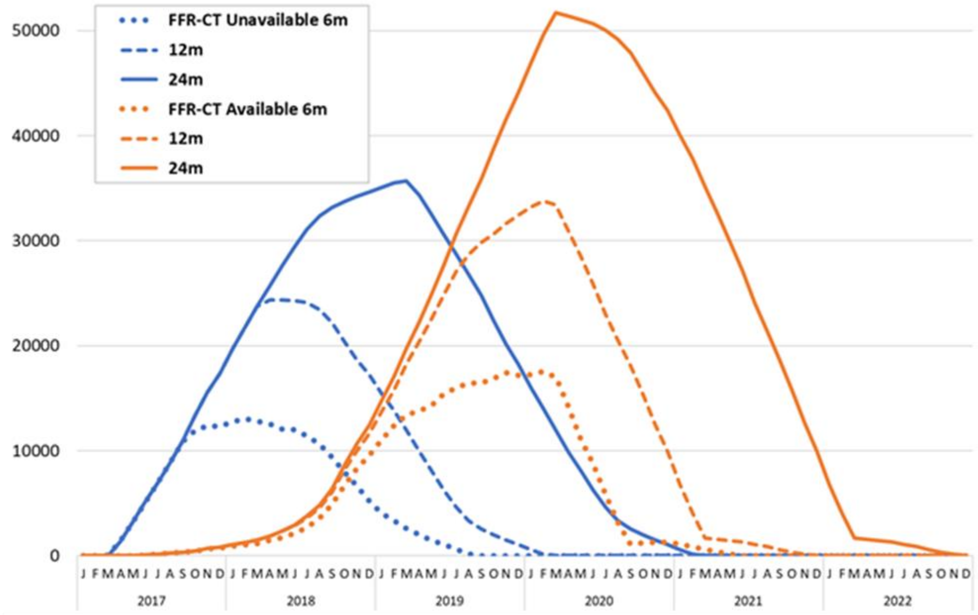

B.

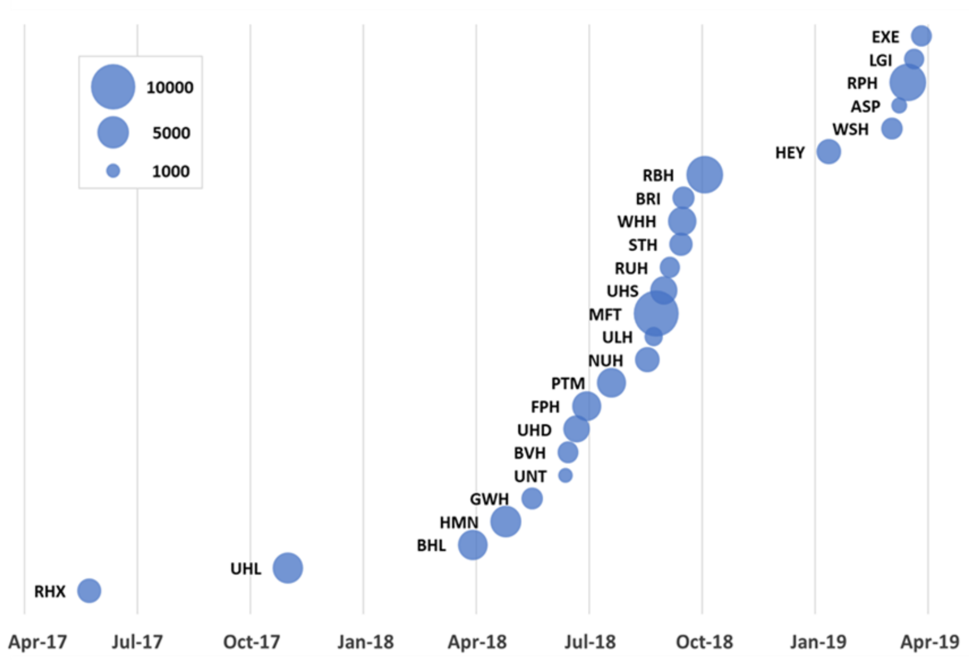

Extended Data Figure 3

A. Myocardial Infarction (MI) events and B. Percutaneous Intervention (PCI) rates at 90 days, 1 year and 2 years.

Cox proportional hazards univariate analysis (unadjusted) p values. The shaded areas indicate the 95% CIs.

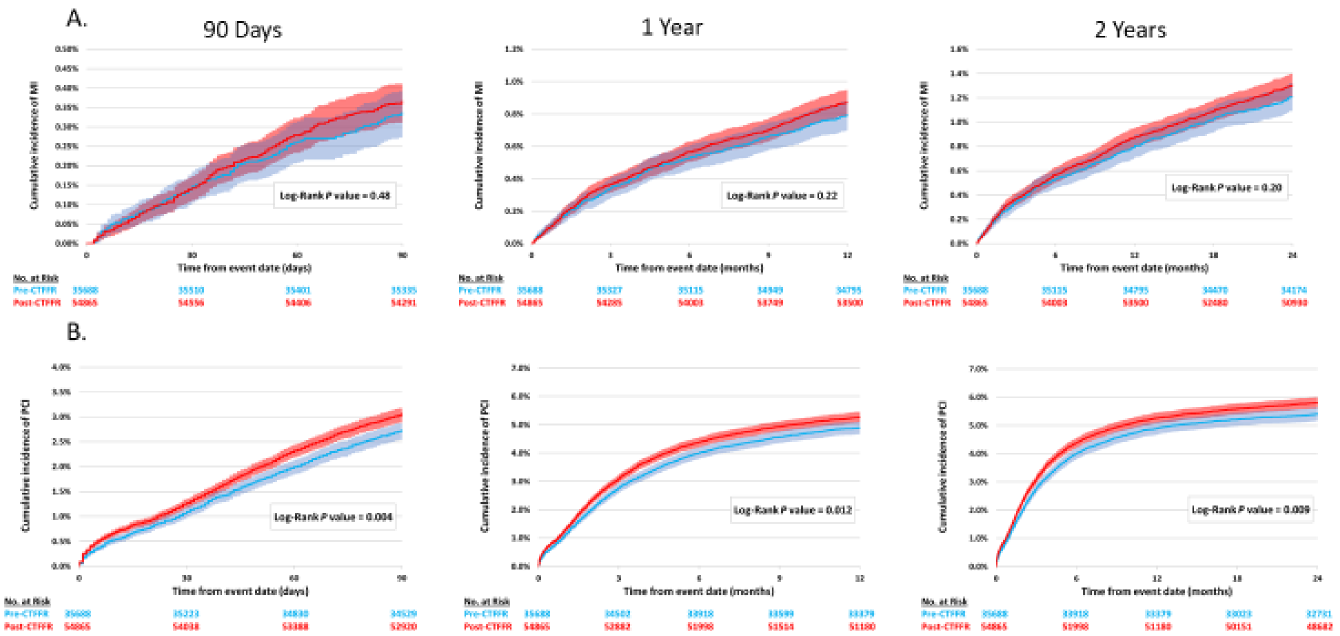

Extended Data Figure 4

Propensity Score Matching covariate balancing pre and post matching using a standard mean difference of <0.05.

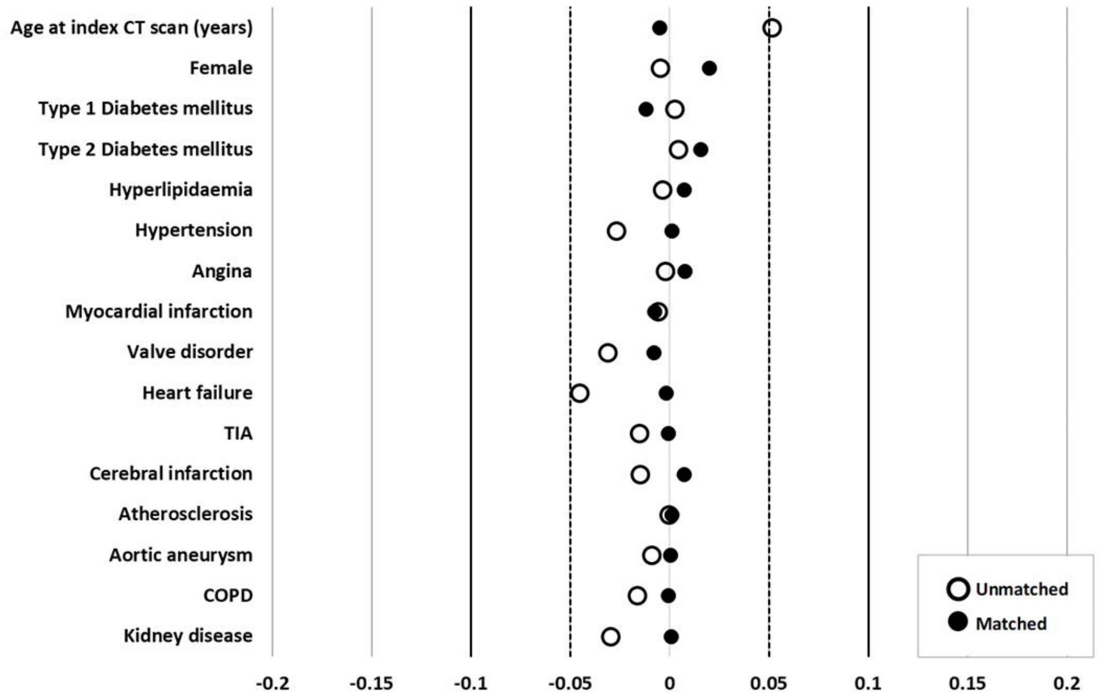

Extended Data Figure 5

Kaplan-Meier (KM) charts of the cumulative incidence of the individual primary objectives over 2 years post index CCTA on the Propensity Matched population (n=30,665 in each group). Blue represents pre FFR-CT availability. Red line represents post FFR-CT availability. The shaded areas indicate the 95% CIs.

A. All-cause death, B. Cardiovascular death, C. Myocardial infarction, D. ICA with no revascularization.

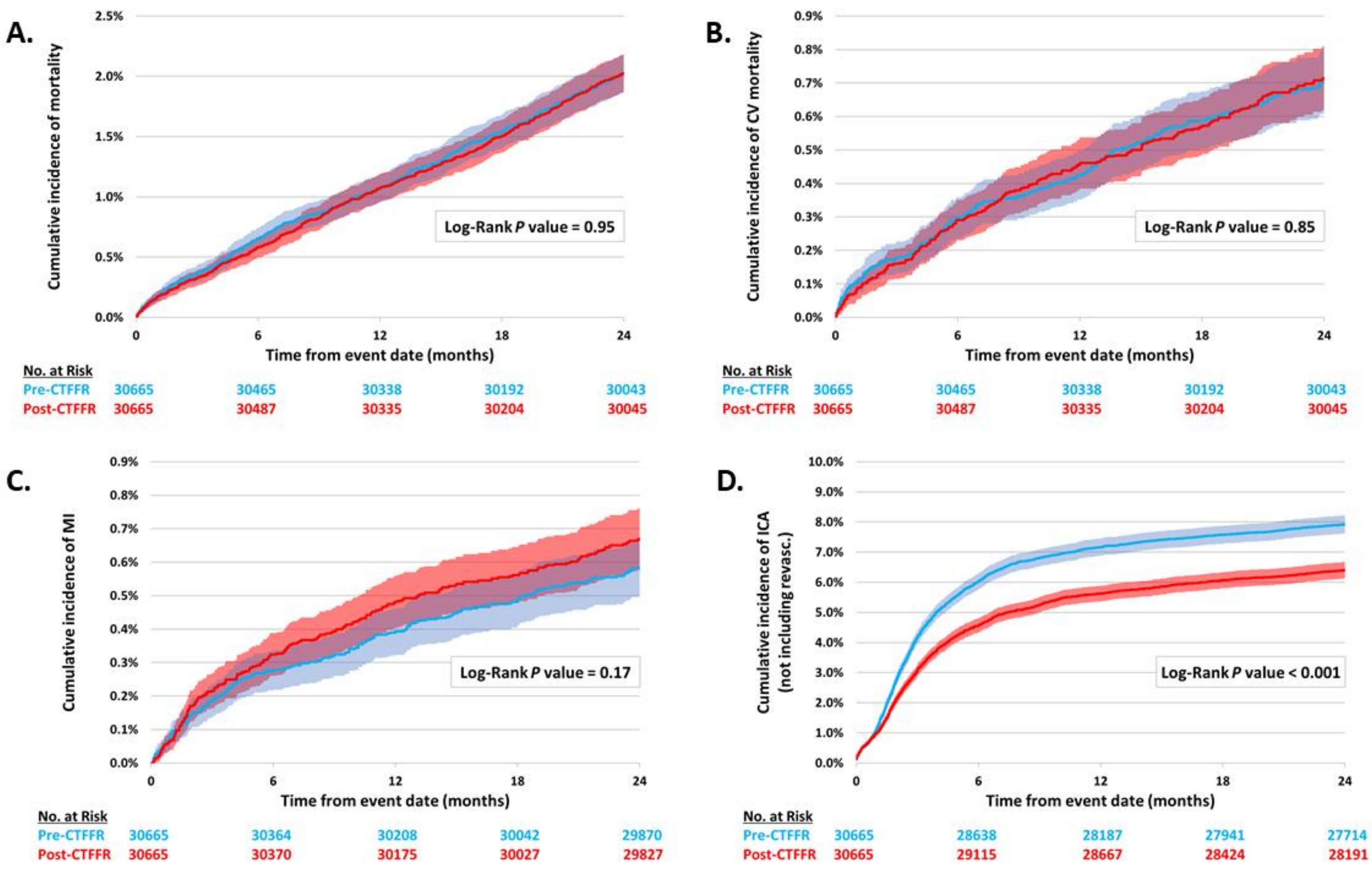

Extended Data Figure 6

Time (in years) from CCTA date (Time 0) to the date of revascularisation (PCI or CABG) for the 6,784 patients who had coronary revascularisation within the 2-year follow-up. The mean wait was 0.41 years for both FFR-CT unavailable and FFR-CT available groups. The group who received FFR-CT had a insignificant shorter wait (0.4 years).

The box represents the interquartile range with the median as the middle segment, the mean as the black dot and the whiskers as minimum and maximum waits.

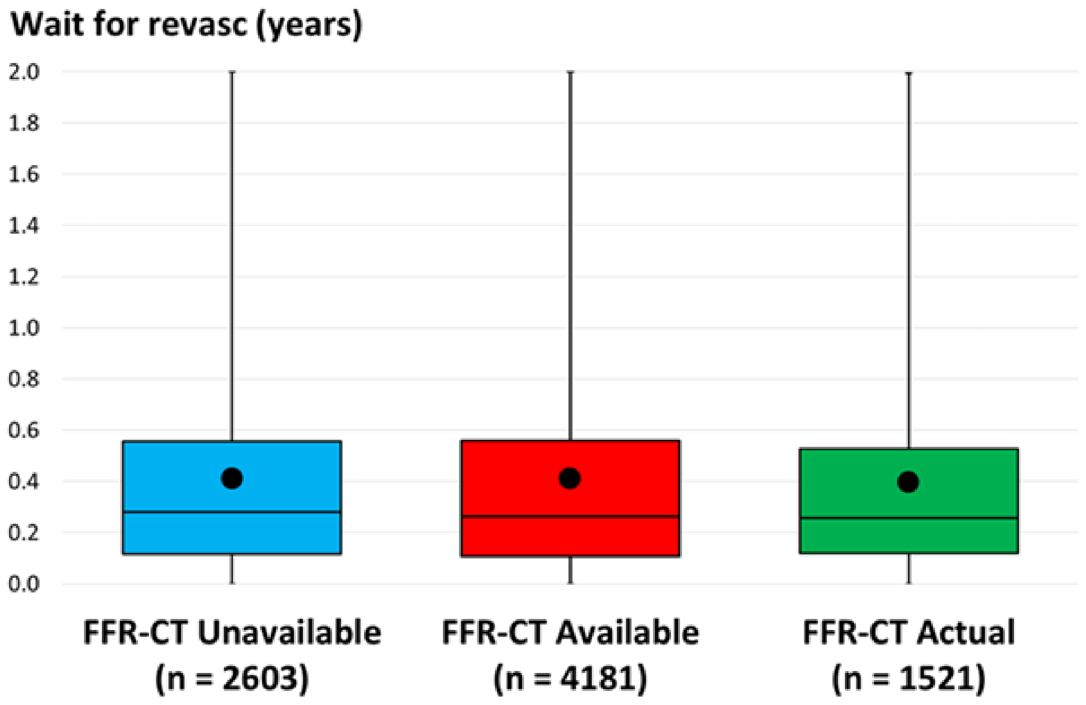

Extended Data Figure 7

Autoregressive Integrated Moving Average (ARIMA) method were performed. Weekly observed rates of all primary outcomes were assessed before and after FFR-CT introduction, then modelled for observed versus expected changes in outcomes post-health intervention over time. The dashed black line represents the time that FFR-CT was made available to the hospital sites.

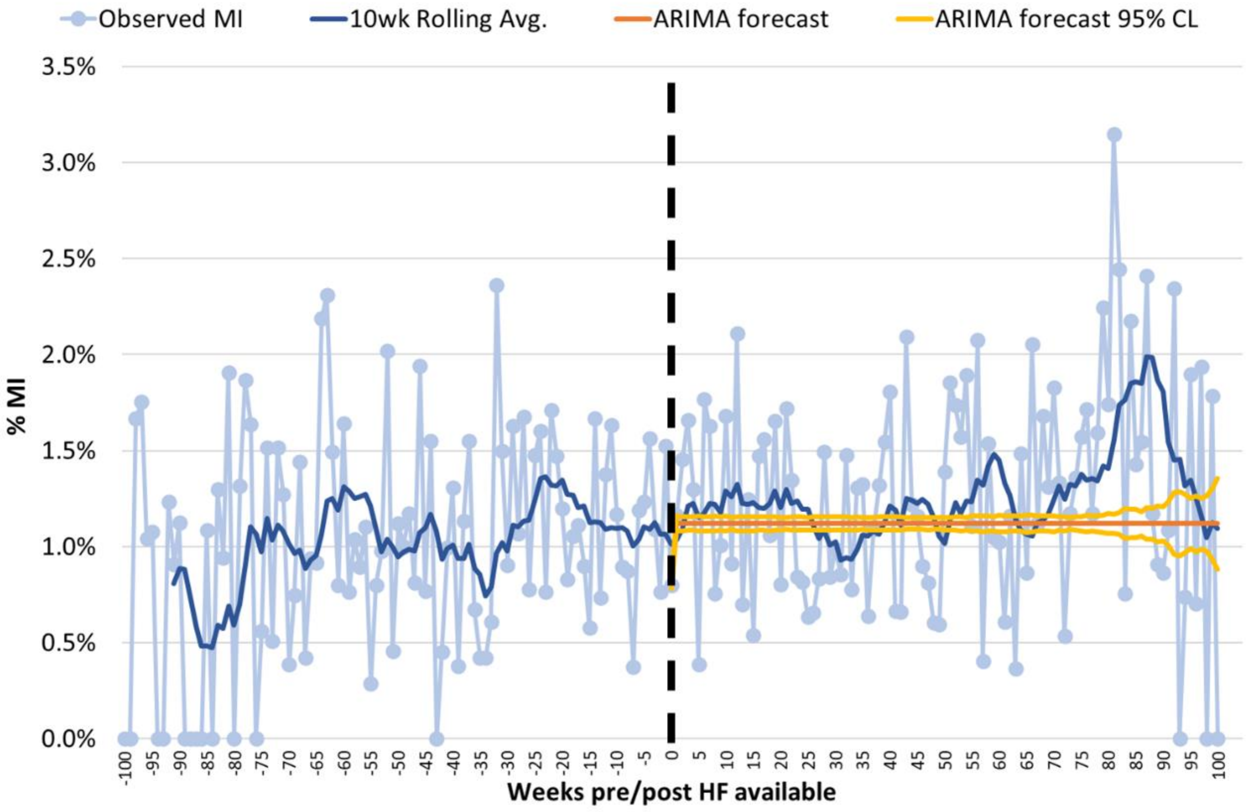

Extended Data Figure 8

A positive FFR-CT (centre panel). The LAD shows a gradual reduction from proximal to distal vessel with a stenosis specific value of 0.72 and distal vessel value of 0.65. The circumflex was ‘negative’ at 0.89 and the RCA was occluded. The scale on the left panel shows the degree of flow limitation and likelihood of functional significance as a continuum with the margins of error on the right panel to help the physician in their decision process.

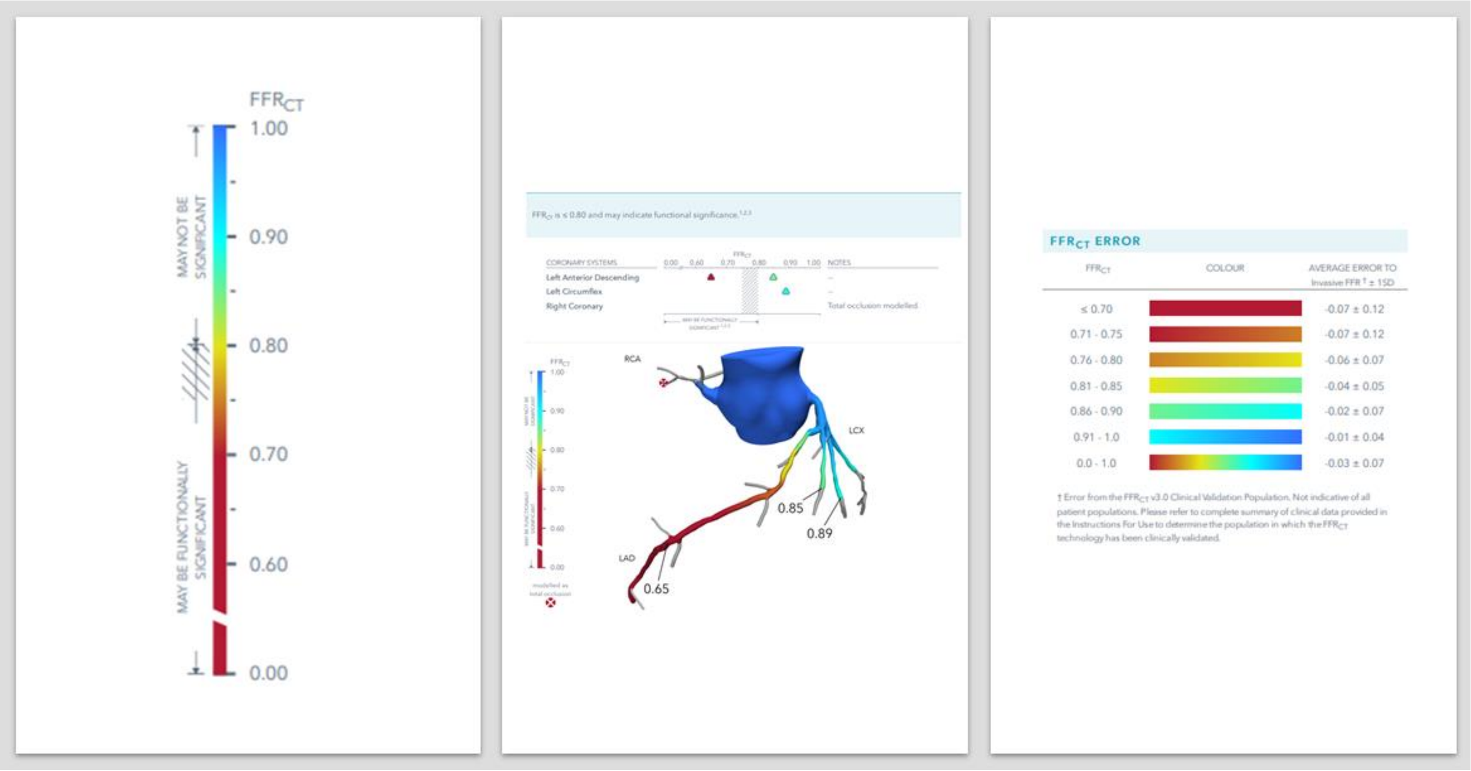

The FISH&CHIPS Protocol

FFRCT In Stable Heart disease and Coronary Computed Tomography Angiography Helps Improve Patient care and Societal costs

Study Objective	The primary objective of FISH and CHIPS is to identify differences in health-related events, time to diagnosis and overall healthcare costs of a stable chest pain population undergoing Coronary Computed Tomography Angiography (CCTA) and Fractional Flow Reserve (FFR_{CT}), compared to a previous 'standard of care' diagnostic chest pain pathway of CCTA and non-invasive functional testing.
Study Design	This is a multi-centre, retrospective, observational analytic cohort study design. The study will utilise the electronic health record (EHR) data already collected by NHS England on all patients that underwent a CCTA for the assessment of coronary artery disease over a 3-year period (April 2017-April 2020). All patients were treated in accordance with the latest NICE clinical guidance (CG 95 2016). Healthcare data will be collected from 6 months prior to and 12 months following the index CCTA. Hospital admissions data collected will include inpatient hospital admissions, outpatient visits, cardiovascular diagnostic tests and procedures. All subsequent clinical events including myocardial infarction and all-cause death will be measured as clinical outcomes. Costs are determined from the NHS national tariff system.
Study Principal Investigator	Dr Timothy Fairbairn<br>Liverpool Heart and Chest Hospital and the University of Liverpool,<br>Thomas Drive,<br>Liverpool,<br>L14 3PE<br>United Kingdom
Sponsor	Liverpool Heart and Chest Hospital,<br>Thomas Drive,<br>Liverpool,<br>L14 3PE<br>United Kingdom

RESEARCH REFERENCE NUMBERS

**IRAS Number:**	285996
**ISRCTN Number / Clinical trials.gov Number:**	ISRCTN57392292
**FUNDERS Number:**	MR/T024933/1

Investigator Protocol Signature Page

The undersigned confirm that the following protocol has been agreed and accepted and that the Chief Investigator agrees to conduct the trial in compliance with the approved protocol and will adhere to the principles outlined in the Medicines for Human Use (Clinical Trials) Regulations 2004 (SI 2004/1031), amended regulations (SI 2006/1928) and any subsequent amendments of the clinical trial regulations, GCP guidelines, the Sponsor's (and any other relevant) SOPs, and other regulatory requirements as amended.

I agree to ensure that the confidential information contained in this document will not be used for any other purpose other than the evaluation or conduct of the clinical investigation without the prior written consent of the Sponsor. I agree to allow the University of Liverpool monitors and auditors and their designees full access to all medical records at the research facility for participants entered in the study. I agree to comply with NHS England's information governance alliance (IGA) General Data Protection Regulation (GDPR) guidance.

I also confirm that I will make the findings of the trial publically available through publication or other dissemination tools without any unnecessary delay and that an honest accurate and transparent account of the trial will be given; and that any discrepancies and serious breaches of GCP from the trial as planned in this protocol will be explained.

For and on behalf of the Trial Sponsor:

Signature: 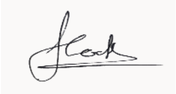

Name: (please print): Jennifer Crooks Deputy Director of Research and Innovation

Chief Investigator:

Date: 22/6/22

Signature: 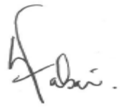

Name: (please print): Dr Timothy Fairbairn

Key Trial Contacts

**Chief Investigator**	Dr Timothy Fairbairn

	t: 0151 600 1477<br>e: timothy.fairbairn@lhch.nhs.uk
**Trial Co-ordinator**	Mr Ian Kemp<br>t: 0151 228 1158<br>e: ian.kemp@lhch.nhs.uk
**Sponsor**	Liverpool Heart and Chest Hospital NHS Trust<br>Thomas Drive,<br>Liverpool,<br>L14 3PE<br>United Kingdom<br>t: 0151 600 1467<br>e: jennifer.crooks@lhch.nhs.uk
**Joint-sponsor(s)/co-sponsor(s)**	NA
**Funder(s)**	Medical Research Council (Funders number MR/T024933/1)<br>and Heartflow
**Clinical Trials Unit**	Liverpool Heart and Chest Clinical Trials \ monitoring officer<br>t: 0151 600 1013<br>e: Colette.mcgee@lhch.nhs.uk
**Key Protocol Contributors**	Dr Timothy Fairbairn, Chief Investigator<br>Professor Rod Stables, CTU lead
**Committees**	Liverpool Heart and Chest hospital Research and Ethics Committee,<br>Liverpool Health Partners (LHP) Single Point of Access to Research and Knowledge (SPARK) Joint Research Sponsorship Committee

1 Table of Contents

Key Trial Contacts........23
1 Table of Contents........25
2 Abbreviations........27
3 Summary........28
3.1 Professional Summary........28
3.2 Plain English Summary........29
4 Introduction........30
4.1 Background........30
4.2 Study Rationale........30
5 Study Objectives........31
5.1 Primary objective........31
5.2 Secondary objective........31
5.3 Primary Endpoints:........31
5.4 Exploratory Endpoints:........31
5.4.1 Qualitative Assessment........32
5.4.2 Imaging biomarkers........32
6 Study Design........32
7 Study Population........33
7.1 Setting and Target Population........33
7.2 Inclusion Criteria........34
7.2.1 Site eligibility:........34
7.2.2 Individual eligibility:........34
7.3 Exclusion Criteria........34
7.4 Follow up........34
8 Statistical Methodology........35
8.1 Sample size and Power calculation........35
8.2 Statistical Analysis........36
9 Good Clinical Practice........36
9.1 Ethical Conduct........36
9.1.1 Informed Consent........36
9.1.2 Good Clinical Practice (GCP)........37
9.2 Data Management and Confidentiality........37
9.2.1 Data Collection........37

9.2.2 FFRCT Data 37
9.2.3 Trial Management 37
10 References 38

2 Abbreviations

CABG	Coronary Artery Bypass Grafting
CAD	Coronary Artery Disease
CCTA	Coronary Computed Tomography Angiography
CEC	Clinical Events Committee
DS	Degree Stenosis
FFR	Fractional Flow Reserve
FFR_{CT}-	CCTA-derived fractional flow reserve
ICA	Invasive Coronary Angiography
IHD	Ischemic Heart Disease
IRB	Institutional Review Board
LAD	Left Anterior Descending coronary artery
LCX	Left Circumflex coronary artery
LMS	Left Main Stem coronary artery
MACE	Major adverse cardiovascular events
MI	Myocardial Infarction
MT	Medical treatment
PCI	Percutaneous coronary intervention
RCA	Right coronary artery
SE	Stress echo
SPECT	Single-Photon Emission Tomography

3 Summary

3.1 Professional Summary

Protocol Title	FFRCT In Stable Heart disease & CCTA Helps Improve Patient care and Spending
Investigation strategy	CCTA plus FFRCT reduces healthcare resource utilization and costs compared to a CCTA strategy alone.
Study Principal Investigator	Dr Timothy Fairbairn
Academic Research Organization	Liverpool Centre for Cardiovascular Science (LCCS), Liverpool Heart and Chest Hospital, Liverpool, UK
Sponsor	The University of Liverpool
Participants and Study sites	Approximately 100,000 patients from 25 sites who received a CCTA for the assessment of CAD in NHS England.
Study Planned Duration	36 months
Primary study objective	To determine whether a CCTA and FFRCT diagnostic pathway reduces health-related events, time to diagnosis and overall healthcare costs compared to a 'standard of care' CCTA diagnostic chest pain pathway.
Primary hypothesis	The addition of FFRCT into a CCTA diagnostic pathway will be safe with no difference in major adverse cardiovascular event rates or death whilst reducing the time to diagnosis, result in fewer downstream tests and reduce overall costs to the healthcare system.
Population	Chest pain patients with suspected stable coronary artery disease being clinically investigated with a CCTA in England.
Study Design and Methods	A pragmatic 'real world' multi-centre, retrospective, observational analytic cohort study design. All patients receiving a CCTA at institutions utilising FFRCT as part of NHS England's Innovation and Technology Payment (ITP) programme. Participants will be recruited from 1 year pre-ITP and the 2 years of the ITP programme. Patients will be followed up at a minimum of 24-months post CCTA for the pre-defined primary and secondary endpoints.
Primary Endpoint	Primary and secondary outcomes measured:<br>1. MI event rate, hospitalization for acute coronary syndrome, MI deaths and all-cause death.<br>2. Downstream testing: numbers of non-invasive functional tests, and invasive coronary angiograms

	without revascularisation performed following the index FFRCT.<br>3. Cost analysis: Total cost to the NHS of the index test and all downstream investigations and hospital admissions.
Secondary Endpoint	1. Time to diagnosis- Trust Referral to Treatment (RTT) time.<br>2. Qualitative assessment of the impact of the FFRCT health technology
Study follow up	Participants will be followed up to a minimum of 24 months

3.2 Plain English Summary

Chest pain may be a symptom that is related to a narrowing of the heart blood vessels (coronary artery disease [CAD]). This chest pain, known as angina, can result in a reduced quality of life and, if not diagnosed and managed appropriately, could result in a heart attack. Coronary disease remains the largest cause of death in the United Kingdom today, with one death every 4 minutes. Guidelines recommend the use of tests to help diagnose and manage chest pain 'angina' patients. Coronary computed tomography angiography (CCTA) is a test that takes images of the heart blood vessels. It is the main test for patients presenting with angina, as it is excellent at saying when the heart blood vessels are normal and can be reassuring for patients. However, when narrowing's are present CCTA lacks the ability to tell whether they are causing the patient's symptoms.

A new technology, CT-derived fractional flow reserve (FFRCT) uses the CCTA images to make a 3D model of the heart blood vessels that shows whether there is a limitation in the blood flow to the heart which is causing the symptoms. The National Institute for Health and Care Excellence (NICE) recommends the use of FFRCT in a chest pain pathway. However, use of this new technology remains limited due to funding restrictions and uncertainty as to its benefit in the NHS.

This study aims to determine the extent to which the new FFRCT technology is safe and reliable, provides a quicker time to diagnosis for the patient, reduces the need for further tests and thus does the investment in the test represent good value to the NHS.

4 Introduction

4.1 Background

The investigation of suspected stable coronary artery disease (CAD) should primarily be based on a non-invasive strategy (Knuuti *et al.*, 2020). In the United Kingdom Coronary Computed Tomography Angiography (CCTA) is now recommended as the first-line diagnostic test for patients with suspected angina and no prior CAD. (NICE, 2010) This recommendation by the National Institute for Health and Care Excellence (NICE) in 2016 was primarily driven by the very high sensitivity of CCTA to detect the presence or absence of coronary atheroma. (Nielsen et al., 2014; Meijboom et al., 2008) Given that the majority of patients with suspected angina turn out to have non-cardiac chest pain, and the majority of CCTA scans performed for this purpose show only minimal or no CAD, a significant proportion of patients can be immediately reassured by CCTA in the current NICE guidelines pathway. (Fordyce, Newby, & Douglas, 2016) However, in approximately a third of cases, CAD detected by CCTA is either indeterminate due to dense calcification or of intermediate severity which results in only modest specificity of CCTA to detect functionally significant 'ischaemic' CAD - and this remains its Achilles' heel. (Meijboom et al., 2008)

Recent advances in technology allow the use of raw CCTA images with computational fluid dynamic modelling to produce a 3D haemodynamic representation of the coronary tree flow limitation. (Lee et al., 2018) (Conte et al., 2017) (Taylor, Fonte, Min, City, & Angeles, 2013) This CT-Derived Fractional Flow Reserve (FFRCT) has developed rapidly since first concept and is now used in routine clinical practice. NICE, in a medical technology guidance (MTG 32), stated 'the clinical and cost effectiveness evidence justified FFRCT's use as a second line functional test for indeterminate or intermediate coronary stenoses'. The guidance also commented that 'based on the current evidence, using HeartFlow FFRCT may lead to cost savings of £214 per patient. By adopting this technology the NHS in England may save a minimum of £9.1 million by 2022 through avoiding invasive investigation and treatment'.

4.2 Study Rationale

The accurate diagnosis of CAD is important to allow the appropriate medical treatment and post-test risk stratification to identify patients that might benefit from revascularisation. FFR_{CT} is a non-invasive physiological test that can assess flow limitation across a coronary stenosis with high diagnostic accuracy and good correlation to invasive FFR.(Nørgaard *et al.*, 2017) FFR_{CT} has been shown in trials to reduce the total number of inappropriate invasive coronary angiograms (ICAs) post-CCTA, by reducing the number of cases with no obstructive coronary artery disease. This increases the revascularization treatment rate, which represents a more efficient use of the expensive catheter angiography laboratory.(Douglas *et al.*, 2015)' (Jensen *et al.*, 2017)' (Nørgaard *et al.*, 2014) Patients could therefore be receiving the test with the highest accuracy, improving diagnostic certainty, thereby reducing unnecessary downstream tests and the time to treatment. The NHS should benefit by reducing the number of invasive tests and the wider economy will benefit from fewer lost working days. In addition, the ITP has allowed national coverage of FFR_{CT} which has the potential to remove regional variations in clinical practice and spending costs.

The existing evidence for the use of FFRCT is based on randomised controlled trials, registry studies and economic analysis from a US providers' perspective. There is no real-world comparative data, and no literature from the perspective of NHS practice, which differs from the more 'invasive' approach in the US. This research will answer whether an NHS FFRCT pathway is better for the patients in terms of safety, reducing unnecessary alternative tests and time to treatment compared to previous 'standard of care' diagnostic pathways (including CCTA alone, stress echocardiography, stress perfusion MRI and nuclear scintingraphy). The impact on the NHS will be determined by comparing costs of a CCTA and selective FFRCT pathway to those of a standard of care pathway as well as the number of hospital visits.

5 Study Objectives

5.1 Primary objective

The study aims to identify differences in health-related events, time to diagnosis and overall costs in a clinical population undergoing CCTA and FFR_{CT} for symptoms suggestive of stable CAD, compared to a previous 'standard of care' diagnostic chest pain pathway.

5.2 Secondary objective

5.3 Primary Endpoints:

1. Safety: Has the implementation of FFR_{CT} been safe?

   End points: Myocardial infarction event rate, hospitalization for acute coronary syndromes and mortality (all-cause and cardiovascular).

2. Time to Diagnosis: Does FFR_{CT} reduce the time to diagnosis and treatment?

   End points: Trust Referral to Treatment (RTT) time.

3. Downstream testing: Does FFR_{CT} reduce the number of downstream investigations and the number of overall invasive and non-invasive diagnostic tests?

   End points: numbers of non-invasive functional tests, and invasive coronary angiograms without revascularisation performed following the index FFR_{CT}.

4. Cost analysis: Does the technology represent value for money?

   End point: Total cost to the NHS of the index test, all downstream investigations, hospital admissions and outpatient visits.

5.4 Exploratory Endpoints:

5.4.1 Qualitative Assessment

A qualitative survey of clinicians at the NHS trusts implementing a FFRCT pathway will be performed to assess the impact of a change in the service aligned with FFRCT. Factors assessed will include: ease of implementation (governance and IT), user friendliness, ease of clinical integration and practicality.

5.4.2 Imaging biomarkers

Using the list of CCTA originally provided by the sites, the participating centres PACS teams will send the anonymised CCTA datasets to the CTU data storage for future linkage to the outcome data provided by NHS Digital. This process will ensure that anonymity is preserved while providing for a valuable resource in terms of a large database of anonymised imaging datasets with outcome data. There will also be the opportunity to repeat the data capture from NHS Digital in future years to establish medium and long-term outcomes. The purpose of creating this repository of anonymised outcome and imaging data is to allow for future research projects into image analysis of CTA including deep learning algorithms, radiomics analysis, and biomechanical modelling of coronary arteries all with the goal to improving future risk stratification to better target therapeutic interventions.

6 Study Design

This is a multi-centre, retrospective, observational analytic cohort study design.

This pragmatic 'real-world' trial, is designed to utilise big data to answer practical health questions and determine clinical outcomes in a timely fashion. Randomized clinical trials (RCT's) in comparative effectiveness research (CER) have been considered the gold standard. These are however, subject to several problems, including cost, patient selection bias and slow translation of knowledge into practice. (Angus, 2015) This study design removes any patient treatment heterogeneity effect seen in RCT's by assessing the impact of a new intervention on a whole population. (Longford, 1999) by utilising the electronic health record (EHR) data already collected by the NHS. This trial will thus represent a true assessment of the effectiveness of a new health technology on a population basis in the current NHS system and will enable the rapid translation of research into clinical and health care policy decisions.

Participants will include all individuals who had a CCTA performed at an institute participating in the NHSE FFRCT ITP during 2018-2020. All CCTA 12 months prior to and up to 24 months following the start of a FFRCT programme (total study period 36 months) will be assessed. The cohort from this population that received an FFRCT will be separately identified, with linkage to the NHS digital data outcomes. HeartFlow will provide FFRCT-specific data.

NHS Digital's Data Access Request Service (DARS) will be queried to provide the patient episodes over the study period. NHSD collects national data sets containing details of all admissions, accident and emergency (A&E) attendances, and outpatient appointments at NHS hospitals in England. DARS will extract data from the following data sets:

- Emergency Care Data Set (ECDS)
- Hospital Episode Statistics Admitted Patient Care (HES APC)

- Hospital Episode Statistics Critical Care (HES CC)
- Hospital Episode Statistics Outpatients (HES OPC)
- Hospital Episode Statistics Accident and Emergency (HES AE)
- Diagnostic Imaging Dataset (DID)
- Medicines Dispensed in Primary Care Data Set (from NHS Business Authority)
- Civil Registration Deaths

These data sets will provide the following information:

- Patient demographics (such as age group, gender and ethnicity)
- Administrative information (such as dates and methods of admission and discharge)
- Geographical information; such as where patients are treated and the area where they live (post code).
- Medications (type, dose and whether processed)
- ICA and revascularisation data linked to patients.
- Incidence of downstream testing; stress echocardiograms, stress MRI or nuclear scintigraphy.

Costs will be calculated for all hospital attendances, diagnostic investigations and treatments from the published NHS England National Prices and Tariff workbook (2017-2019- HRG/OPEC codes) with the appropriate market forces factor applied. Health economic modelling will be performed by the University of Liverpool.

Referral to treatment (RTT) data is collected by each NHS trust for each patient. This data will be collected by the local research teams.

7 Study Population

7.1 Setting and Target Population

The study population will include all patients that received a CCTA for symptoms suggestive of CAD at a participating institute, 12 months prior to the institutes first FFRCT study and up to 24 months following (total study period up to 36 months). The timeframe of study recruitment is dependent upon the starting time of each centre in the ITP programme. Centres that had a later start date (on-boarding) will have a shorter recruitment period, with a minimum of 12 months. These centres represent the 'real-world' NHS hospitals that are a mixture of secondary and tertiary referral centres (not dedicated academic or research sites) with clinical experience in CCTA who have met the minimum quality standards set out by NHS England. Thus, the study population represents a true reflection of the current CCTA practice in the national population, reducing the potential effect of selection bias seen in many RCT's.

A study population of 85,292 patients received either CCTA analysis alone (standard of care group, n=75,361) or CCTA and Heartflow FFRCT analysis (FFRCT group, n=9,799) during the two years of the new technology being available to each site. The total study population over the 36 months is likely to be over 100,000.

7.2 Inclusion Criteria

7.2.1 Site eligibility:

- Sites within NHS England with a FFRCT ITP programme commencing between April 1st 2018-March 31st 2020
- Sites must have performed a minimum number of ≥50 FFRCT within 1 year of their programme commencing

7.2.2 Individual eligibility:

- Age ≥18 years
- CCTA for the assessment of coronary artery disease (CAD)

7.3 Exclusion Criteria

- Age <18 years
- Coronary artery calcium scoring alone
- CCTA in addition to a second CT investigation for a non-coronary indication (CT TAVI, CT aorta)
- Previous CCTA within 6 months
- Prior CABG / MI
- Entry into a separate FFRCT research study during the study timeframe

7.4 Follow up

Patient data will be collected up to a minimum of 24 months post-CCTA. Clinical data will also be analysed for the 6 months pre-CCTA to ensure no cross over between diagnostic pathways and prior testing (including CCTA) within 6 months (Figure 1).

Longitudinal long-term follow-up (>2 years) at 5 and 10 years would be feasible and cost effective using the same methodology of HES downloads and data analysis. This would provide a true long-term perspective of health care resource use in a stable angina population

Figure 1 Study Design Overview

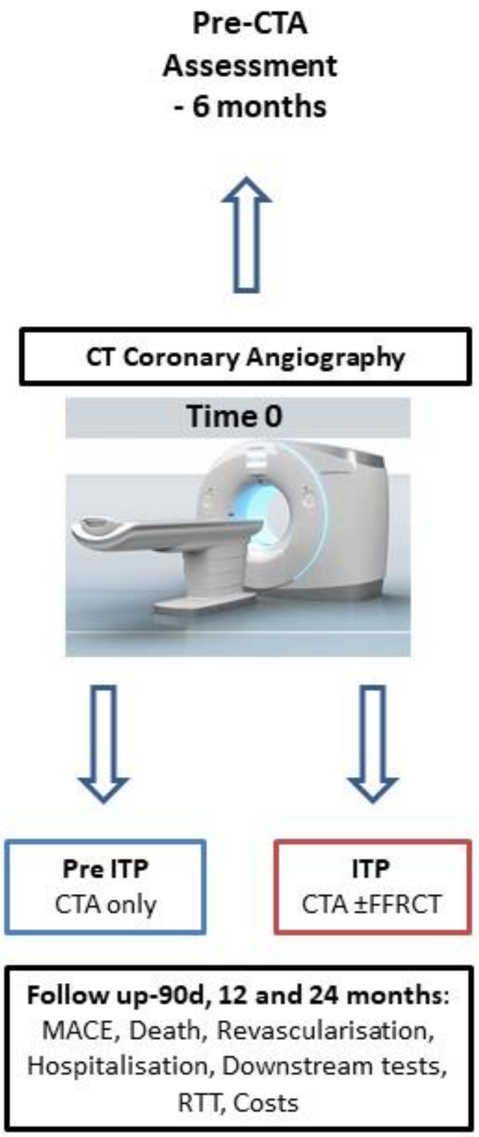

8 Statistical Methodology

8.1 Sample size and Power calculation

As an observational analytic cohort study design, this trial requires no power calculation for estimates of effect. However, multiple previous studies have guided the sample size and estimates of expected clinical outcomes. Disease prevalence at CCTA can be estimated from SCOTHEART (n=4778), where the coronary arteries were normal in 37%, non-obstructive CAD in 38% and

obstructive CAD in 25% of a UK population. The CONFIRM registry study showed in a contemporary US population of over 5000 patients investigated by CCTA that the annual event rate varied between 0.31% for normal coronary arteries to 2.06% in the instance of obstructive CAD (Leipsic et al., 2013). The international ADVANCE registry study of a patient population being investigated with FFRCT had cardiovascular event rates of 1.16% at 1 year. Thus, it is possible to estimate expected clinical outcome event rates and compare to actual observed events across the pathways to determine the safety of a UK CCTA pathway.

8.2 Statistical Analysis

Analysis of the primary end-point is based on the rate of adverse events (MACE) as a composite of all-cause death, myocardial infarction and invasive coronary angiography without revascularization. Event rates over time will be calculated using Kaplan-Meier methodology from the time of the CCTA. Cox proportional hazard ratios will be used to determine the odds ratio (OR) of receiving revascularization post FFRCT compared to other tests.

Time to diagnosis will be compared using an ‘intention to diagnose analysis’ by analysing groups according to their investigative test (FFRCT vs CCTA alone).

The primary cost analysis will include total patient pathway costs at 12 months, with comparison between the two testing strategies. The mean cost difference with 95% confidence intervals and P value will be calculated. Sub-analysis will categorise the total costs breakdown as; Investigations, hospital stay, procedural costs.

Cost sensitivity analyses will be applied to the modelling, looking at different cost utilities in the UK and regional variability.

9 Good Clinical Practice

9.1 Ethical Conduct

The study will be conducted in accordance with the principles of Good Clinical Practice (GCP). Ethical approval will be sought from the Health Regulatory Authority (HRA), the trial will comply with the principles set out in the declaration of Helsinki and the UK policy framework for health and social care research.

9.1.1 Informed Consent

As a retrospective study we will be accessing confidential patient information without consent in England. Therefore, consent was approved on the basis of health and social care research in the public interest (National Health Service Act 2006 -s251 - 'Control of patient information'. , through an application to the Confidentiality Advisory Group (CAG) and ethical approval from the Health Regulatory Authority (HRA)

Patient information will be kept to a minimum needed for the purposes of the research project and will be kept securely for the duration of the study and up to 15 years post study completion. Data will be linked to health records using the NHS number by NHS Digital. All research sites and the clinical trials unit will comply with the UK General Data Protection Regulation (GDPR).

9.1.2 Good Clinical Practice (GCP)

Members participating in the study will be encouraged to complete their GCP training.

9.2 Data Management and Confidentiality

9.2.1 Data Collection

The data will be collected from 25 NHS trusts participating in the NHS England ITP FFRCT programme, a life science industry (HeartFlow) and the national data collection institute (NHS Digital).

The sponsor and principal investigators are responsible for the handling, processing, accuracy and quality assurance of data collection. The study teams will be familiar with the study protocol and requirements. Data will be recorded in a confidential manner. The study staff will comply with the Data Protection Act 2018 with regards to collection, storage, processing and disclosure of data. All data will be stored within the NHS trust framework with password protection and external server backup. The legal basis for processing data will be based on the General Data Protection Regulation Article 6 (1) (e) and General Data Protection Regulation Article 9 (2) (j).

Publication of the study results will not include any patient identifiable data. Data will be archived and stored for 15 years.

9.2.2 FFRCT Data

Patient data was anonymised prior to sending to HeartFlow as part of the clinical service in accordance with local and national clinical governance regulations. Data linkage to the hospital episodes statistics will be performed using a non-identifiable, anonymised methodology. Patient data will remain anonymised and personal information will remain in the hands of NHS organisations.

9.2.3 Trial Management

The study will be conducted by a team of researchers including the principal investigator, co-investigators and a dedicated trial team at Liverpool Heart and Chest Hospital Clinical Trials Unit (CTU). A Trial Steering Committee will be formed and study oversight will be co-ordinated by the sponsors' research committee, with quarterly progress reports. All records will be made available to the sponsor and ethics committee for review or as part of an audit of the study.

10 References

Angus, D. C. (2015). Fusing randomized trials with big data: The key to self-learning health care systems? JAMA - Journal of the American Medical Association, 314(8), 767–768.

BHF. (2018). UK Factsheet. British Heart Foundation, https://ww(August).

Collet, C., Onuma, Y., Andreini, D., Sonck, J., Pompilio, G., Mushtaq, S., … Serruys, P. W. (2018). Coronary computed tomography angiography for heart team decision-making in multivessel coronary artery disease. European Heart Journal, 39(41), 3689–3698.

Conte, E., Annoni, A., Pontone, G., Mushtaq, S., Guglielmo, M., Baggiano, A., … Andreini, D. (2017). Evaluation of coronary plaque characteristics with coronary computed tomography angiography in patients with non-obstructive coronary artery disease : a long-term follow-up study. European Heart Journal Cardiovascular Imaging, 18, 1170–1178.

Curzen, N. P., Nolan, J., Zaman, A. G., Norgaard, B. L., & Rajani, R. (2016). Does the Routine Availability of CT – Derived FFR In fl uence Management of Patients With Stable Chest Pain Compared to CT Angiography Alone? JACC. Cardiovascular Imaging, 9(10), 1188–1194.

Danad, I., Van Rossum, A. C., Knaapen, P., Van De Ven, P. M., Van Royen, N., Raijmakers, P. G., … Taylor, C. A. (2018). 1185Head-to-head comparison of FFR-CT against coronary CT angiography and myocardial perfusion imaging for the diagnosis of ischaemia. European Heart Journal, 39(suppl_1).

Douglas, P. S., Pontone, G., Hlatky, M. A., Patel, M. R., Norgaard, B. L., Byrne, R. A., … De Bruyne, B. (2015). Clinical outcomes of fractional flow reserve by computed tomographic angiography-guided diagnostic strategies vs. usual care in patients with suspected coronary artery disease: the prospective longitudinal trial of FFRct: outcome and resource impacts stud. European Heart Journal, 36(47), 3359–3367.

Fairbairn, T. A., Nieman, K., Akasaka, T., Nørgaard, B. L., Berman, D. S., Raff, G., … Patel, M. R. (2018). Real-world clinical utility and impact on clinical decision-making of coronary computed tomography angiography-derived fractional flow reserve : lessons from the ADVANCE Registry. European Heart Journal, 0(August), 1–11.

Fordyce, C. B., Newby, D. E., & Douglas, P. S. (2016). Diagnostic Strategies for the Evaluation of Chest Pain. Journal of the American College of Cardiology, 67(7), 843–852.

Hlatky, M. A., De Bruyne, B., Pontone, G., Patel, M. R., Norgaard, B. L., Byrne, R. A., … Douglas, P. S. (2015). Quality-of-Life and Economic Outcomes of Assessing Fractional Flow Reserve with Computed Tomography Angiography: PLATFORM. Journal of the American College of Cardiology, 66(21), 2315–2323.

Izzat, L., & Knight, E. (2011). Copyright Medinews (Cardiology) Limited Reproduction Prohibited Copyright Medinews (Cardiology) Limited Reproduction Prohibited Copyright Medinews (Cardiology) Limited Reproduction Prohibited Copyright Medinews (Cardiology) Limited Reproduction Pr. British Journal of Cardiology, 18(2), 2009–2012.

Jensen, J. M., Bøtker, H. E., Mathiassen, O. N., Grove, E. L., Øvrehus, K. A., Pedersen, K. B., … Nørgaard, B. L. (2017). Computed tomography derived fractional flow reserve testing in stable patients with typical angina pectoris : influence on downstream rate of invasive coronary angiography. European Heart Journal Cardiovascular Imaging, 0, 1–10.

Knuuti, J. *et al.* (2020) '2019 ESC guidelines for the diagnosis and management of chronic coronary syndromes', *European Heart Journal*, 41(3), pp. 407–477.

Lee, S.-E., Sung, J. M., Rizvi, A., Lin, F. Y., Kumar, A., Hadamitzky, M., & Kim, Y.-J. (2018). Quantification of Coronary Atherosclerosis. Circulation. Cardiovascular Imaging, 14(July), 1–10.

Leipsic, J., Taylor, C. M., Heilbron, B. G., Mancini, G. B. J., Achenbach, S., Berman, D. S., … Min, J. K. (2013). Cardiovascular Risk among Stable Individuals Suspected of Having Coronary Artery Disease with No Modifiable Risk Factors : Results from an International Multicenter Study of 5262 Patients 1. Radiology, 267(3), 718–726.

Longford, N. T. (1999). Selection bias and treatment heterogeneity in clinical trials. Statistics in Medicine, 18(12), 1467–1474.

Lu, M. T., Ferencik, M., Roberts, R. S., Lee, K. L., Ivanov, A., Adami, E., … Hoffmann, U. (2017). Noninvasive FFR Derived From Coronary CT Angiography: Management and Outcomes in the PROMISE Trial. JACC: Cardiovascular Imaging, 10(11), 1350–1358.

Meijboom, W. B., Van Mieghem, C. A. G., van Pelt, N., Weustink, A., Pugliese, F., Mollet, N. R., … de Feyter, P. J. (2008). Comprehensive Assessment of Coronary Artery Stenoses. Computed Tomography Coronary Angiography Versus Conventional Coronary Angiography and Correlation With Fractional Flow Reserve in Patients With Stable Angina. Journal of the American College of Cardiology, 52(8), 636–643.

Min, J. K., Leipsic, J., Pencina, M. J., Berman, D. S., Koo, B. K., Van Mieghem, C., … Mauri, L. (2012). Diagnostic accuracy of fractional flow reserve from anatomic CT angiography. JAMA - Journal of the American Medical Association, 308(12), 1237–1245.

Nakazato, R., Park, H. B., Berman, D. S., Gransar, H., Koo, B. K., Erglis, A., … Min, J. K. (2013). Noninvasive fractional flow reserve derived from computed tomography angiography for coronary lesions of intermediate stenosis severity results from the DeFACTO study. Circulation: Cardiovascular Imaging, 6(6), 881–889.

NICE. (2010). Chest pain of recent onset: assessment and diagnosis. NICE Guidance, (March 2010). Retrieved from https://www.nice.org.uk/guidance/cg95/chapter/guidance

Nielsen, L. H., Ortner, N., Nørgaard, B. L., Achenbach, S., Leipsic, J., & Abdulla, J. (2014). The diagnostic accuracy and outcomes after coronary computed tomography angiography vs. conventional functional testing in patients with stable angina pectoris: a systematic review and meta-analysis. European Heart Journal Cardiovascular Imaging, 15(9), 961–971.

Nørgaard, B. L., Hjort, J., Gaur, S., Hansson, N., Bøtker, H. E., Leipsic, J., … Jensen, J. M. (2017). Clinical Use of Coronary CTA–Derived FFR for Decision-Making in Stable CAD. JACC: Cardiovascular Imaging, 10(5), 541–550.

Nørgaard, B. L., Leipsic, J., Gaur, S., Seneviratne, S., Ko, B. S., Ito, H., … Achenbach, S. (2014). Diagnostic performance of noninvasive fractional flow reserve derived from coronary computed tomography angiography in suspected coronary artery disease: The NXT trial (Analysis of Coronary

Blood Flow Using CT Angiography: Next Steps). Journal of the American College of Cardiology, 63(12), 1145–1155.

Nørgaard, B. L., Terkelsen, C. J., Mathiassen, O. N., Grove, E. L., Bøtker, H. E., Parner, E., … Jensen, J. M. (2018). Coronary CT Angiographic and Flow Reserve-Guided Management of Patients With Stable Ischemic Heart Disease. Journal of the American College of Cardiology, 72(18), 2123–2134.

Rajani, R., Webb, J., Marciniak, A., & Preston, R. (2015). Comparative efficacy testing - Fractional flow reserve by coronary computed tomography for the evaluation of patients with stable chest pain. International Journal of Cardiology, 183, 173–177.

Roel S. Driessen, MDa, Ibrahim Danad, MDa, Wijnand J. Stuijfzand, MDa, Pieter G. Raijmakers MD, PhDb, Stefan P. Schumacher, MDa, Pepijn A. van Diemen, MDa, Jonathon A. Leipsic, Md., Juhani Knuuti, MD, PhDd, S. Richard Underwood, MD, PhDe, Peter M. van de Ven, P., & Albert C. van Rossum, MD, PhDa, Charles A. Taylor, PhDg, Paul Knaapen, MD, P. (2019). Comparison of Coronary Computed Tomography Angiography, Fractional Flow Reserve, and Perfusion Imaging for Ischemia Diagnosis. Journal of the American College of Cardiology, (2), 161–173.

Stewart, S., Murphy, N., Walker, A., McGuire, A., & McMurray, J. (2003). The current cost of angina pectoris to the National Health Service in the UK. Heart, 89(8), 848–853.

Taylor, C. A., Fonte, T. A., Min, J. K., City, R., & Angeles, L. (2013). Computational Fluid Dynamics Applied to Cardiac Computed Tomography for Noninvasive Quantification of Fractional Flow Reserve Scienti fi c Basis. Journal of the American College of Cardiology, 61(22), 2233–2241.

Williams, M. C., Hunter, A., Shah, A. S. V, Assi, V., Lewis, S., Smith, J., … Newby, D. E. (2016). Use of Coronary Computed Tomographic Angiography to Guide Management of Patients with Coronary Disease. Journal of the American College of Cardiology, 67(15), 1759–1768.

FISH and CHIPS Analysis plan

Background

Evaluation of the implementation of a health technology (Heartflow CT FFR) into the health system in NHS England. The FAC study encompasses all sites that were eligible and utilised the technology in its first year of roll out (2018). This represents a complex health intervention as it includes over 27 sites and 24 integrated care boards (ICB) across NHS England.

The aim of FAC is to determine the practical effectiveness of the complex health technology intervention on the system as a whole. Comparative analysis will differentiate between groups where the technology was not available (usual care) to times that the technology was available (new care) for clinical and cost effectiveness.

Primary Analysis

Primary Endpoints:

1. MI event rate, MI deaths and all-cause death.
2. Invasive coronary angiogram without revascularization
3. Downstream testing: numbers of non-invasive functional tests, and invasive coronary angiograms without revascularisation performed following the index CTA
4. Cost analysis: Total cost to the NHS of the index test and all downstream investigations, hospital outpatient visits and hospital admissions.

Event rates of the composite outcome of MI, MI death or all cause death over-time will be calculated using Kaplan-Meier methodology from the time of the CCTA at 12 and 24 months. Stratification and multiple regression techniques will be used to address confounding variables with adjusted Odds Ratio (OR) used to determine clinical outcomes of 'usual care' versus 'new care'. The odds ratio (OR) of receiving angiography and revascularization post FFRCT compared to other tests will be determined. 95% confidence intervals (CI) will estimate the precision of the OR.

Interrupted Time Series (ITS) methods will be used to evaluate the impact of the health intervention. Intercorrelation pre and post intervention phases will be used and the effect metrics of a change in level versus a change in slope will be determined. The design of the ITS includes; Time interval (e.g. monthly); total number of observations; total number of time intervals; average number of observations per time interval;

Time to diagnosis will be compared using an 'intention to diagnose analysis' by analysing groups according to their investigative test (FFRCT vs CCTA alone).

Comment: For MACE events this will be analysed on first event basis rather than cumulative. For DIDs test will be on a cumulative basis.

Secondary Endpoints:

1. Time to diagnosis- Trust Referral to Treatment (RTT) time.
2. Qualitative assessment of the impact of the FFRCT health technology

The primary cost analysis will include total patient pathway costs at 24 months, with comparison between the two testing strategies. The mean cost difference with 95% confidence intervals and P value will be calculated. Sub-analysis will categorise the total costs breakdown as; Investigations, hospital stay, procedural costs.

Cost sensitivity analyses will be applied to the modelling, looking at different cost utilities in the UK and regional variability.

Interrupted Times Series Analysis

Study characteristics: Author name, year of publication, rationale for using an ITS design, and type and description of the intervention or

exposure.

Design Time interval (monthly), total number of observations before aggregation, total number of time intervals (n=60), and 2 segments (using the beginning time for each site entering the ITP programme), number of time intervals per segment, average number of observations per time interval and that there is a comparison group (FFRCT group).

Outcome Description: MI events, MI death, all cause death, ICA and revascularization rate and count of the outcomes at the individual observation level and description of the aggregate-level outcome (e.g., rate per population).

Model: Model shape (level change AND slope change); model type (ARIMA, segmented regression, other regression, and pre-post); modelling approach for any transition period; roll in implementation period of first 50 cases of CT FFR

Statistical methods: Statistical estimation method (e.g., logistic regression); with autocorrelation and outliers were investigated; and how they were handled in the analysis (Praise Winston correction); whether and how no stationarity was tested for.

Effect measures: Reported effect measures (e.g., change in level and change in slope), whether an absolute or relative measure, effect estimates and statistics associated with the effect measure (e.g., P values and confidence intervals), details on any forecasting (e.g., projecting from one segment to a specified time point in another segment), and whether there was mention of any ceiling or floor effects.
