# Supplementary material for: Implementation of a national AI technology program on cardiovascular outcomes and the health system
Source: Nat Med. 2025 Apr 4;31(6):1903–10. doi: 10.1038/s41591-025-03620-y (PMC12176617; doi:10.1038/s41591-025-03620-y)
Supplement: Supplementary file 2 — Reporting Summary [file 41591_2025_3620_MOESM2_ESM.pdf]

nature portfolio

Corresponding author(s): Dr Timothy Fairbairn, Associate Professor University of Liverpool, Cardiologist Liverpool Heart and Chest Hospital

Last updated by author(s): Feb 5, 2025

Reporting Summary

Nature Portfolio wishes to improve the reproducibility of the work that we publish. This form provides structure for consistency and transparency in reporting. For further information on Nature Portfolio policies, see our Editorial Policies and the Editorial Policy Checklist.

Statistics

For all statistical analyses, confirm that the following items are present in the figure legend, table legend, main text, or Methods section.

n/a	Confirmed	
☐	☒	The exact sample size (*n*) for each experimental group/condition, given as a discrete number and unit of measurement
☐	☒	A statement on whether measurements were taken from distinct samples or whether the same sample was measured repeatedly
☐	☒	The statistical test(s) used AND whether they are one- or two-sided<br>*Only common tests should be described solely by name; describe more complex techniques in the Methods section.*
☐	☒	A description of all covariates tested
☐	☒	A description of any assumptions or corrections, such as tests of normality and adjustment for multiple comparisons
☐	☒	A full description of the statistical parameters including central tendency (e.g. means) or other basic estimates (e.g. regression coefficient) AND variation (e.g. standard deviation) or associated estimates of uncertainty (e.g. confidence intervals)
☐	☒	For null hypothesis testing, the test statistic (e.g. *F*, *t*, *r*) with confidence intervals, effect sizes, degrees of freedom and *P* value noted<br>*Give P values as exact values whenever suitable.*
☒	☐	For Bayesian analysis, information on the choice of priors and Markov chain Monte Carlo settings
☒	☐	For hierarchical and complex designs, identification of the appropriate level for tests and full reporting of outcomes
☐	☒	Estimates of effect sizes (e.g. Cohen's *d*, Pearson's *r*), indicating how they were calculated

Our web collection on statistics for biologists contains articles on many of the points above.

Software and code

Policy information about availability of computer code

Data collection	No software was used.
Data analysis	The underlying algorithms for FFRct analysis have been previously published (Taylor, Charles A., et al. "Patient-specific modeling of blood flow in the coronary arteries." Computer Methods in Applied Mechanics and Engineering 417 (2023): 116414.); however, the source code is proprietary and used for commercial purposes, so it cannot be made available publicly. All statistical analysis was performed in R stats package (R documentation, version 3.6.2).

For manuscripts utilizing custom algorithms or software that are central to the research but not yet described in published literature, software must be made available to editors and reviewers. We strongly encourage code deposition in a community repository (e.g. GitHub). See the Nature Portfolio guidelines for submitting code & software for further information.

Data

Policy information about availability of data

All manuscripts must include a data availability statement. This statement should provide the following information, where applicable:

- Accession codes, unique identifiers, or web links for publicly available datasets
- A description of any restrictions on data availability
- For clinical datasets or third party data, please ensure that the statement adheres to our policy

All data was collected from NHS digitals Data Access Reporting System (DARS) databases. Supporting data is available at the UK Data service repository (https://

reshare.ukdataservice.ac.uk) including study Protocol and the algorithms for defining clinical outcomes from HES data. Dataset availability is subject to controlled access due to the Confidentiality Advisory Group (CAG) approvals and NHS England's Data Sharing contract (CON-317153-H1H4Z (Version 2.03)). Individual de-identified, aggregated participant data that underlie the study reported outcomes will be made available in accordance with the ethical approvals, NHSE data sharing framework contract and the Medical Research Council Industrial Collaboration Agreement (MICA).
Any data sharing is subject to a Data Sharing Agreement (DSA) between parties. Each DSA will detail:
I. the Data to be provided;
II. the legal basis for sharing Data;
III. the Purpose of the sharing and use of the Data;
IV. the expected benefits to health and/or social care by sharing the Data;
V. the data transfer method;
VI. any Associated DSAs;
VII. any special terms and conditions for the use or reuse of the Data; and
VIII. any Charges payable for the provision of the Data.
Requests for data sharing should be communicated in writing to the research governance team at Liverpool Heart and Chest Hospital (Research.Governance@lhch.nhs.uk) specifying the nature of the request. All external requests will be responded within 2 weeks by the sponsors director of research, with an estimated timeframe of 3 months from the date of request to DSA approval. The study ethics approvals allow for data storage up to 15 years.

Research involving human participants, their data, or biological material

Policy information about studies with human participants or human data. See also policy information about sex, gender (identity/presentation), and sexual orientation and race, ethnicity and racism.

Reporting on sex and gender	Sex based analysis have been performed and reported
Reporting on race, ethnicity, or other socially relevant groupings	Race, ethnicity and social groupings (as defined by the UK Indices of Social Deprivation) have been performed and reported in the results section and supplemental material. 78.7% white British or Irish, 2.2% Black, 1.4% mixed race, 8.2% Asian, 2.4% other, 7.1% unstated. Mean IMD score 20.
Population characteristics	The final study population of 90,553 patients was divided into 35,688 CCTA prior to the introduction of FFR-CT, and 54,865 CCTA after FFR-CT was available at their hospital. The mean age was 58±13 years, 48.1% female.
Recruitment	All patients who received a CCTA scan from April 2017-Dec 2020 were included. As a retrospective observational cohort study, the Confidentiality Advisory Group (CAG) approved the use of confidential patient information without consent on the basis of health and social care research in the public interest (National Health Service Act 2006 -s251 - 'Control of patient information'). The NHSE opt out of research database was queried with participants excluded if consent was withdrawn. Between April 2017-December 2020, 102,616 CCTA were performed. There were 289 (0.28%) patients without an NHS number, 5,674 (5.5%) patients withdrew their consent, 6,100 (5.9%) CCTA were repeat studies on the same patient during the study period and 20 (0.0001%) patients had a post-mortem CCTA. The final study population was 90,553 patients.
Ethics oversight	The Confidentiality Advisory Group (CAG) approved the use of confidential patient information without consent on the basis of health and social care research in the public interest (National Health Service Act 2006 -s251 - 'Control of patient information'; CAG Reference 20CAG0101). Ethical approval was obtained from the Health Regulatory Authority (HRA), (IRAS project ID 285996; REC reference:20/NW/0430). The NHSE opt out of research database was queried with participants excluded if consent was withdrawn. The study was performed in accordance with the Declaration of Helsinki and principles of Good Clinical Practice (GCP).

Note that full information on the approval of the study protocol must also be provided in the manuscript.

Field-specific reporting

Please select the one below that is the best fit for your research. If you are not sure, read the appropriate sections before making your selection.

☒ Life sciences ☐ Behavioural & social sciences ☐ Ecological, evolutionary & environmental sciences

For a reference copy of the document with all sections, see nature.com/documents/nr-reporting-summary-flat.pdf

Life sciences study design

All studies must disclose on these points even when the disclosure is negative.

Sample size	As an observational analytic cohort study design, this trial requires no power calculation for estimates of effect. However, multiple previous studies have guided the sample size and estimates of expected clinical outcomes. Disease prevalence at CCTA can be estimated from SCOTHEART (n=4778), where the coronary arteries were normal in 37%, non-obstructive CAD in 38% and obstructive CAD in 25% of a UK population. The CONFIRM registry study showed in a contemporary US population of over 5000 patients investigated by CCTA that the annual event rate varied between 0.31% for normal coronary arteries to 2.06% in the instance of obstructive CAD (Leipsic et al., 2013). The international ADVANCE registry study of a patient population being investigated with FFRCT had cardiovascular event rates of 1.16% at 1 year. Thus, it is possible to estimate expected clinical outcome event rates and compare to actual observed events across the pathways to determine the safety of a UK CCTA pathway.
Data exclusions	No data exclusions

Replication	No findings were replicated as this is an observational cohort study
Randomization	Retrospective, observational cohort study. The final study population of 90,553 patients was divided into 35,688 CCTA prior to the introduction of FFR-CT, and 54,865 CCTA after FFR-CT was available at their hospital
Blinding	Blinding did not occur during patient recruitment/scanning as this was an observational cohort study. The study analysis required the investigators to know whether the patients had their CCTA prior to or after the introduction of the AI technology at their hospital site.

Reporting for specific materials, systems and methods

We require information from authors about some types of materials, experimental systems and methods used in many studies. Here, indicate whether each material, system or method listed is relevant to your study. If you are not sure if a list item applies to your research, read the appropriate section before selecting a response.

Materials & experimental systems

n/a	Involved in the study
☒	☐ Antibodies
☒	☐ Eukaryotic cell lines
☒	☐ Palaeontology and archaeology
☒	☐ Animals and other organisms
☐	☒ Clinical data
☒	☐ Dual use research of concern
☒	☐ Plants

Methods

n/a	Involved in the study
☒	☐ ChIP-seq
☒	☐ Flow cytometry
☒	☐ MRI-based neuroimaging

Clinical data

Policy information about clinical studies
All manuscripts should comply with the ICMJE guidelines for publication of clinical research and a completed CONSORT checklist must be included with all submissions.

Clinical trial registration	ISRCTN Number / Clinical trials.gov Number: ISRCTN57392292
Study protocol	Final protocol is published on the study website hosted at Liverpool heart and Chest Hospital Clinical Trials Unit
Data collection	All patients having CCTA at 27 NHS England hospitals from April 2017-December 2020. Clinical events were determined from routinely collected healthcare data from April 2016- April 2022. This provided 6 years of event data with a median 1 year prior and 3.3 years follow up post-CCTA. Data was requested pre-CCTA to ensure accurate baseline patient characterisation. Data from all hospital episodes, including diagnostic coding, were obtained from NHS Digital's Data Access Reporting System (DARS). This comprised the Hospital Episode Statistics (HES) Admitted Patient Care (APC), Critical Care (CC), Emergency Care (ECDS) and Outpatient Care (OPC) datasets. Diagnostic tests performed were captured in the Diagnostic Imaging Dataset (DIDS). Mortality data and cause of death was obtained from the Office for National Statistics (ONS) linked HES dataset. FFR-CT analysis was performed by a commercial company (HeartFlow Inc., Mountain View, US)
Outcomes	Differences in health-related events between the populations were used to determine the primary objectives of safety and impact of implementing FFR-CT to the health system. Primary safety outcomes included all-cause mortality, cardiovascular mortality and myocardial infarction (MI) event rates. Primary impact outcomes included the rates of downstream tests performed following the index CCTA; including ICA with and ICA without revascularisation as well as non-invasive functional tests or repeat CCTA. Secondary analysis categorised FFR-CT patients' outcomes according to their FFR-CT results.

Plants

Seed stocks	NA
Novel plant genotypes	NA
Authentication	NA
